# Metabolomics analyses and comparative insight to neuroprotective potential of unripe fruits and leaves of *Citrus aurantium* ethanolic extracts against cadmium-induced rat brain dysfunction: involvement of oxidative stress and akt-mediated CREB/BDNF and GSK3β/NF-κB signaling pathways

**DOI:** 10.1007/s11011-024-01513-6

**Published:** 2025-01-06

**Authors:** Doaa A. H. Deabes, Eman A. W. El-Abd, Sara M. Baraka, Zeinab A. El-Gendy, Reda M. S. Korany, Marwa M. Elbatanony

**Affiliations:** 1 Pharmacognosy Department, National Research Centre (NRC), El Behouth St., P.O. 12622, Cairo, Egypt; 2 Chemistry of Natural Compounds Department, National Research Centre, Giza, 12622 Egypt; 3 Department of Pharmacology, Medical Research and Clinical Studies Institute, National Research Centre, Dokki, Giza, Egypt; 4 Pathology Department, Faculty of Veterinary Medicine, Cairo University, Giza, Egypt

**Keywords:** *Citrus aurantium*, Flavonoids, Cadmium, Neurotoxicity, Cognitive function

## Abstract

Serious neurological disorders were associated with cadmium toxicity. Hence, this research aimed to investigate the potential neuroprotective impacts of the ethanolic extracts of *Citrus aurantium* unripe fruits and leaves (CAF and CAL, respectively) at doses 100 and 200 mg/kg against cadmium chloride-provoked brain dysfunction in rats for 30 consecutive days. HPLC for natural pigment content revealed that CAF implied higher contents of Chlorophyll B, while the CAL has a high yield of chlorophyll A and total carotenoid. Fifty-seven chromatographic peaks were identified by UPLC/MS**/**MS; 49 and 29 were recognized from CAF or CAL, respectively. Four compounds were isolated from CAF: 3’,4’,7 –trihydroxyflavone, isorhainetin, vitexin, and apigenin. *In vitro* studies outlined the antioxidant capacity of studied extracts where CAF showed better scavenging radical DPPH activity. Results clarified that both extracts with a superior function of CAF at the high adopted dose significantly ameliorated CdCl_2_-induced neuro-oxidative stress and neuro-inflammatory response via restoring antioxidant status and hindering nuclear factor kappa B (NF-κB) stimulation. Moreover, it up-regulated the levels of phospho-protein kinase B (p-Akt), phospho- cAMP-response element binding protein (p-CREB), and brain-derived neurotropic factor (BDNF) levels, and elicited a marked decrease in the content of glycogen synthase kinase 3 beta (GSK3β), besides amending Caspase-3 and hyperphosphorylation of tau protein in brain tissues. Moreover, a significant improvement in the rats’ behavioral tasks of the CAL and CAF-treated groups has been recorded, as indicated by marked preservation in locomotion, exploratory, and memory functions of the experimental rats. In conclusion, the reported neuroprotective impacts of *C. aurantium* extracts may be through modulating p-AKT/p-CREB/BDNF and / or p-Akt/ GSK3β/NF-κB signaling pathways.

**Supplementary Information:**

The online version contains supplementary material available at 10.1007/s11011-024-01513-6.

## Introduction

Cadmium (Cd) is a hazardous non-essential metal designated as a human carcinogen (Bhattacharya 2022). Humans could be exposed to Cd through various sources including occupational risks (Fertilizers, mining works, manufacture of certain batteries, and electroplating process), food and water pollution, and tobacco consumption. Based on the FAO/WHO committee estimations, the average human Cd exposure from the diet was reported to be 25 µg/kg/bw per month (Joint et al. 2016). Cd can accumulate in the kidney, liver, lung, and brain due to its sluggish rate of elimination from the body and long biological half-life (10–30 years) (Peana et al. 2022). Given its ionic and molecular mimicry, Cd enters the bloodstream, infiltrates the blood-brain barrier (BBB), and encounters neurons through voltage-gated calcium passages, causing neurological damage throughout the peripheral nervous system and the central nervous system (CNS) (Zhou et al. 2022).

As Cd reaches neurons, it first affects the endothelium reticulum permeability and consistency; neuron death happens subsequently (Al Olayan et al. 2020). Another mechanism whereby Cd-induced brain injury is through a greater generation of reactive oxygen/nitrogen species (ROS/RNS), which triggers cellular injury and weakens the antioxidant shield mechanism, thereby eventually resulting in neurodegeneration and memory loss (Fan et al. 2021). Indeed, Cd-provoked neurotoxicity has been widely linked to neurodegenerative diseases and psychiatric problems (Namgyal et al. 2021). Cd poisoning exhibited symptoms of depression and anxiety in both experimental rodents and humans (Balali-Mood et al. 2021; Zhao et al. 2021).

Despite the global attention and health organizations’ recommendations to reduce Cd contamination sources, Cd exposure levels in humans and living organisms are still in a growing manner year by year. It is worth noting that there is no specific treatments have been documented in the case of Cd toxicity. Therefore, many techniques have been validated to eliminate or attenuate Cd toxicity by working as chelating agents for this metal and thereby lessening its absorption by tissues, or enhancing its removal by excretion (Mitra et al. 2022). A recent study postulated that antioxidants and anti-inflammatory agents could ameliorate Cd-induced neurotoxicity by amplifying the anti-oxidative capability through the body to diminish the oxidative stress prevalence (Liu et al. 2023).

Natural extracts with various bioactivities, especially those with pharmaceutical potential and natural antioxidant molecules, have attracted more and more attention and research (Randhawa et al. 2021; Sood et al. 2023). Citrus is the most economically important genus in the Rutaceae family, and it includes trees, plants, and shrubs all over the world. The chemical diversity, nutritional worth, and uses of citrus plants in the food industry and cosmetics make this genus with a global significance value (Ben Hsouna et al. 2023). Citrus fruits include lemon (*Citrus limon*), grapefruit (*Citrus paradisi*), the orange (*Citrus sinensis*), and lim (mostly *Citrus aurantifolia*) as well as citron (*Citrus medica* L.) and mandarin (*Citrus reticulata*). In addition to using citrus plants as a food source, it was highly used in folk medicine in treating many health ailments such as respiratory disorders (cough, bronchitis, tuberculosis, and cold), stress-related diseases (hypertension, anxiety, and depression), and even menstrual irregularity (Chaudhari et al. 2016). Moreover, the pharmacological properties of different *Citrus* species have been reported (Dongre et al. 2023; El-Abd et al. 2024; Lu et al. 2023). For example, Pruthi et al. revealed the efficacy of *C. reticulata* var. kinnow leaf extracts against memory dysfunction in mice via modulating the central cholinergic system and lessening oxidative stress (Pruthi et al. 2021). *Citrus aurantium L.* is a citrus species known as sour orange or bitter orange that has commercial significance and is utilized as a medical and nutritional supplement; nevertheless, it is not employed as an edible fruit due to its sour and bitter flavor which is reported due to the presence of naringin and neohesperidin (Karabıyıklı et al. 2014). Given its unique attributes, this species can be easily differentiated from other Citrus cultivars, where it is an evergreen tree that may reach five meters tall with characteristic fragrant white flowers. In addition, its fruit has a distinctive flavor, and it has abundant amounts of phytochemicals which are known to have significant health benefits. It originated in eastern Africa and Syria and has since been cultivated in the United States, Spain, Italy, and Turkey as a substitute for lemon juice in vegetable salads and appetizers (Maksoud et al. 2021). *C. aurantium* has multiple therapeutic potentials. These biological credentials include activities such as anti-bacterial, antioxidant, antidiabetic, anti-cancer, anti-anxiety, and anti-obesity (Suntar et al. 2018). The antioxidant capacity of *C. aurantium* peel and juice was evaluated and could be attributed to its essential oil which is rich in limonene, and the presence of ferulic and *p*-coumaric acids that were recognized as the predominant phenolic compounds of their methanolic extracts (Marzouk 2013). So, *C. aurantium* peel and juice are regarded as a potential source of natural antioxidants for the food sector. Furthermore, the antibacterial and antifungal activity of its essential oils, with linalool and limonene being the predominant components in both leaves and peels was documented (Azhdarzadeh and Hojjati 2016). According to prior studies, the antioxidant and antimicrobial actions of C. *aurantium* leaf extracts have been informed (Nidhi et al. 2020; Tang et al. 2021). A recent study demonstrated the antioxidant properties of *C. aurantium* unripe fruit extract (Luo et al. 2023). Flavonoids, the principal bioactive chemicals present in *C. aurantium*, are classified into three types: flavanones, flavones, and flavonols. Alkaloids such as p-synephrine and limonoids such as limonin and nomilin have also been discovered (Maksoud et al. 2021). Two novel phenolic glycosides; 1-*O*−3, 5-dihydroxyphenyl-(6-*O*−4-hydroxybenzoyl)-*β*-D-glucopyranoside and 1-*O*−3, 5-dihydroxyphenyl-(6-*O*−3-methoxy-4-hydroxy benzoyl)-*β*-D-glucopyransode were isolated from the fruit of *C. aurantium* (Zhang et al. 2017).

Given the pharmacological properties of *C. aurantium*, this study was established to speculate the potential neuroprotective effects of the ethanolic extracts of *C. aurantium* leaves and unripe fruit against Cd-triggered neurotoxicity *in vivo* on the behavioral, molecular, biochemical, and histopathological levels. As it is established that all bioactive plant phytochemicals may be vary due to the stage of maturation and time of collection, hence, we also intended to identify the chemical content of *C. aurantium* unripe fruits and leaves.

## Materials and methods

### Phytochemical study

#### Plant materials

The fresh *C. aurantium* fruits in an unripe stage and leaves were collected from 6^th^. October garden in 2022. The authentication of specimens was kindly validated by Mrs. Theresa Labib, consultant in taxonomy at the Ministry of Agriculture, Egypt. A voucher specimen (M 249) was deposited in the Herbarium of National Research Centre, Giza, Egypt.

#### Solvent extraction

The air-dried powdered (200 g) unripe fruits and (230 g) leaves of *C. aurantium* were extracted separately on cold by petroleum ether and then with ethanol several times. Under reduced pressure at 40º C, the solvent was evaporated to dryness.

#### HPLC for natural pigment content

The ethanol extract (1 g) was homogenized for pigment extraction in stoppered tubes using 90% aqueous methanol solution for each sample. The resulting mixture was centrifuged at 3000 rpm, for 10 min. Measurements were performed in triplicates and determined using Helios α spectrophotometer at 653 and 664 nm, for chlorophyll a and b, respectively, while at 470 nm for carotene as follows:

Chlorophyll a = 15.65 A666–7.340 A653, Chlorophyll b = 27.05 A653–11.21 A666, Carotene = 1000 A470–2.860 Chl a – 129.2 Chl b/245.

The readings were interpreted in mean, SD, and coefficient of variation values (ANOVA software) (Costache et al. 2012).

#### Ultra-performance liquid chromatography coupled with mass spectroscopy (UPLC–ESI–MS/MS) analysis

The analysis of ethanol extract was performed using UPLC–ESI–MS/MS **(**Triple TOF 5600+, Waters) at the metabolomics unit (Children Cancer Hospital (57357), Cairo, Egypt). The C18 column (reversed phase Exion Xbridge) was used for HPLC separation. The utilized solvent systems (mobile phase) were system 1(0.1% formic acid and deionized water), system 2 (1% methanol and ammonium formate buffer, 5 mM, pH 8), and system 3 (acetonitrile, 100%). Systems 1 and 3 were used for the– ion mode, while systems 2 and 3 were used for the + ion mode. The concentration of the injected sample was adjusted to 1 µg/µl. The following conditions were applied: flow rate was 0.3 mL per 60 s at a temperature of 40 °C, from 0 to 60 s, elution was isocratic (90% system 1 or 2, and 10% system 3), after minute 1 till the twenty-five minute, linear gradient from 90 to 10% system 1 or 2, and 10–90% system 3, and from the minute 25.01 till the twenty-eight minute, elution system as in the first minute. System 1 and 2 were applied for – and + ion mode, respectively. Concerning the conditions of mass spectrometric, firstly, for–ion mode: run period was 28 min and 50–1000 Da mass range. Regarding MS1, the flow rates of nitrogen (nebulizer gas), nitrogen, and curtain (drying gas) were 45, 45 & 25 psi, respectively, as well as the temperature was set at 500 °C, and the voltage of ion spray was − 4500 V. Similar conditions have been applied for MS2, with some changes; 80 V de-clustering potential and 35 V & 20 V collision energy spread CE and CES, respectively. To apply the + ion mode, similar conditions have been followed as in the – ion mode with some modifications: the voltage of ion spray, capillary, and end was set at 4500 V, 4000 V and − 500 V, respectively, and the nebulizer gas was nitrogen at 35.0 psi. The scanning range of mass (m/z range) was 120–1000 amu. The amplitude of Te fragmentation was set at 135 eV. MS2 records were assimilated in + ion mode (El Sayed et al. 2020).

####  Isolation of compounds from *C.* *aurantium* ethanol unripe fruit extract

The silica gel column chromatography approach was used on ethanol unripe fruit extract (6 g) utilizing an elution system of gradient concentrations of chloroform/methanol. The concentration of mobile phase: starting 100% chloroform with a gradual increase of polarity with methanol by 20% until reaching 100% methanol. A total of 80 fractions of 100 ml were successively collected. Each fraction was concentrated under a vacuum. Based on Rf values, the ensuing similar fractions were grouped. The purification of isolated compounds was accomplished using TLC silica plates with a system consisting of chloroform/methanol (90:10 v/v) and sprayed with 1% AlCl3 solution.

#### Chemicals

The utilized solvent and chemicals were at a high level of quality. The flavonoids (standard) were supplied from Sigma Aldrich, USA.

#### Instrumentals

The melting point was evaluated using Koffler’s heating stage microscope. UV–Visible Spectrophotometer double beam UVD– 3500, Labomed, Inc., Visible Spectrophotometer, Shimadzu UV 240 (PIN 204–58000) (Japan), Nuclear Magnetic Resonance spectrometers JEOL EX-270 MHz, 300 MHz and 125 MHz for ^1^H-NMR and ^13^C-NMR, rep., Mass spectrometer; Finnigan Model 3200 at 70 eV.

### *In vitro* DPPH radical scavenging assay of tested extracts

The extracts’ ability to scavenge free radicals was assessed using the 2,2-diphenyl-1-picrylhydrazyl (DPPH, Sigma-Aldrich) radical scavenging approach (Blois 1958; Desmarchelier et al. 2005). A volume of 2.4 mL of 0.1 mM DPPH in methanol was combined with 0.8 mL of the extract under investigation at concentrations of 50–1000 µg/mL prepared in methanol. The reaction mixture was shaken before being placed in a dark place at room temperature for exactly half an hour. The absorbance was recorded at 517 nm using a UV-Spectrophotometer (Jasco V-730). A volume of 1.2 mL of DPPH solution combined with 0.8 mL of methanol served as the control. At doses ranging from 1 to 50 µg/mL, quercetin was employed as a control. The percentage of DPPH radical scavenging activity (%Inhibition) was calculated as follows:

$$\%\mathrm{Inhibition}=\left[\left({\mathrm A}_0-{\mathrm A}_{\mathrm S}\right)/{\mathrm A}_0\right]\times100$$

Where, A_0_ and A_S_ are the reading of control and test extract or standard, respectively.

Extrapolation from regression analysis yielded the IC_50_ value (the concentration of sample necessary to bleach 50% of DPPH radicals). The experiments were done 3 times.

### *In vivo* study

#### Chemicals and reagents

Cadmium chloride was obtained from Sigma-Aldrich, MO, USA with high purity equals to 99.9% labeled with CAS Number: 10108-64-2. Nitric acid (HNO_3_) was provided from Merck, Germany. All chemicals and reagents used for conducting the required analyses were of excellent analytical level.

#### Animals and Ethics

Adult male rats (Wistar albino, 180–200 g bw) were bought from the National Research Centre’s Animal House Colony (Dokki, Giza, Egypt). The rats were housed in suitable cages and kept under conventional temperature and humidity settings, as well as 12-hour light/dark cycles. The animals were given a conventional rat pellet diet (21% protein, 3.48% fat, 3.71% fiber, 0.92% calcium, 0.43% phosphorus, and 2450 kcal/kg energy) supplied by a local manufacturer (National Research Centre’s Animal House Colony, Dokki, Giza, Egypt), and free access to drinking water. This work was carried out in compliance with the ethical procedures and policies delineated in the National Institutes of Health guide for the care and use of Laboratory animals (NIH Publications No. 8023, revised 1978)., the ARRIVE guidelines and international legislation, which was approved by the Medical Research Ethics (MREC) Committee of the National Research Centre, Dokki, Giza, Egypt (Approval no. 7441201-2023).

#### Acute toxicity experiment

The toxicological experiments were conducted in accordance with the OECD 423 criteria (Schlede 2002). The experiment began with a beginning dose of 2000 mg/kg body of tested extract weight given to three animals. The rats were fasted for 4 h before the testing material was administered. The extracts under research were taken orally at the prescribed dose, then the rats were fasted for 2 h and mortality was monitored. The studied animals were monitored for death, behavioral abnormalities, and clinical symptoms of toxicity for 30 min after dosage and then every day for 14 days at the same time. In the current investigation, two dosages of each extract were administered: 100 mg/kg/day (Low dose) and 200 mg/kg/day (High dose).

#### Experimental protocol

The rats were kept for a week before the procedures to be acclimatized and then randomly separated into six groups of 6 rats each (*n* = 6), as follows: **Group I** (Control): the rats were given dis. water orally for 30 days. **Group II** (CdCl_2_): rats were orally administered CdCl_2_ at a dosage of 5 mg/kg bw per day for 30 days, the dosage of CdCl_2_ (< median lethal dose) was selected based on a prior study of Abdel-Aleem and Khaleel (2018) who used the same dose to establish CdCl_2_-induced neurotoxicity in rats for 30 days. **Groups III**,** and IV** (CAL 100 + CdCl_2_ & CAL 200 + CdCl_2_, respectively): for a month, the animals were given CAL at dose 100 or 200 mg/kg rat bw respectively via gastric tube then after 1 h, CdCl_2_ was administered as in group II. **Groups V**,** and VI** (CAF 100 + CdCl_2_ & CAF 200 + CdCl_2_, respectively): rats in these groups received a daily oral dosage of 100 and 200 of CAF respectively followed by an intra-gastric administration of CdCl_2_ same as in the second group by 1 h interval for 30 days. The CdCl_2_ and the tested extracts were dissolved in dis. water at the adopted concentrations and orally administered.

After the last administration (24-h interval) on day 31 of the experiment, the rodents were subjected to behavioral tests. Under anesthesia (24-h later of the behavioral assessments) that was accomplished using pentobarbital (0.3 g/kg b.w/ip), the rats were decapitated and their brains were instantly dissected out and divided on ice into 4 pieces as follows: the brain was cut to two hemispheres, and then each hemisphere was longitudinally sectioned into two parts. One piece of the brain tissue was homogenized in a 0.05 M phosphate buffer (pH 7) to produce a 10% (w/v) tissue homogenate using a tissue Homogenizer acquired from Biospec Product, mini-BeadBeater-8, USA. The homogenate was centrifuged at 10,000 rpm for 20 min at 4 °C using a cooling centrifuge (Laborezentrifugen, 2k15, Sigma, Germany) to remove cell debris, nuclei, erythrocytes, intact cells and mitochondria. The obtained supernatants were separated and kept at −80 ºC for the biochemical assays. The total protein content in brain homogenate was quantified following the method of Bradford (1976). Other part was placed in 10% neutral-buffered formalin for histological and immunohistochemical examination. The residual portions were maintained at − 80 °C for molecular studies and Cd determination.

#### Behavioral tests

##### Motor activity

Using Activity cage (M 7420; Ugo Basile, Italy), the rat spontaneous motor activity was counted. On the 29th day of the study, the animals were allowed to acclimatize at room temperature for 60 min. The activity was assessed using the traditional infrared photocell approach, with any interruption being captured. A total of photocell disruptions were recorded for each 4 min interval period (Marazioti et al. 2005).

##### Open field task

In a square wooden box divided into sixteen equal squares (20 × 20 cm / square) with a height reaching 40 cm, the open field task was carried out. For 5 min, each rat’s locomotor activity was recorded. The box has been cleaned after each rat to eradicate any possible bias created by scents from other rats or to avoid inaccurate conclusions from olfactory signs. The rat ambulation frequency (sum of squares traveled), rearing (sum of times that the rat stands on its hind limbs), and grooming (sum of times that the rat itched its face with its hind limbs or licked its forelimbs) were all recorded for each animal (Walsh andCummins 1976).

##### Y-maze task

This test was performed to evaluate rats’ short-term memory (Kraeuter et al. 2019). The Y-maze is a wooden maze with 3 arms that form the letter Y. The following measures apply to each arm: 50 cm in length, 25 cm in height, and 10 cm in width. It was attached to a central platform at a 120 degree angle. Each rat was positioned in the center and given 5 min to freely traverse the maze. To avoid errors caused by olfactory signals, a 70% ethanol solution was used to clean the maze throughout the trials. Visual observation was done to record the sequence of arm entry, followed by the spontaneous modification; SPA (%) was determined per each animal as follows: SPA (%) = [Successive triplet sets (entry into three distinct arms) /total sum of arm entries-2] ×100.

### Cadmium concentration

Each sample’s brain tissue was digested in 5 ml of 65% HNO3 and 1 ml of 30% H_2_O_2_ utilizing Anton-Paar microwave digestion equipment (Multiwave PRO). Inductively Coupled Plasma Optical Emission Spectrometer (ICP-OES) Model Agilent 5100 Synchronous Vertical Dual View (MY15180008) was used for Cd concentration analysis against standard Cd samples provided by Merck Company and a quality control sample obtained from the National Institute of Standards and Technology. The concentration of Cd in the tissue was determined and expressed as ng/g tissue.

### Biochemical analyses

#### Estimation of brain oxidative stress and antioxidant parameters

As a hallmark of oxidative damage to DNA; 8-hydroxydeoxyguanosine (8-OHdG), an oxidized nucleoside of DNA was determined in the brain tissue homogenate using a rat ELISA kit purchased from CUSABIO, CAT. No. CSB-E10526r (Houston, TX 77054, USA). Malondialdehyde (MDA, lipid peroxidation biomarker) was detected in the brain tissue homogenate samples through rat ELISA assay kit acquired from BioVision (CAT. No. #K739-100, Milpitas, CA 95035, USA). On the other hand, the antioxidant activity was assessed by determining reduced glutathione (GSH) utilizing a rat ELISA kit provided by BioVision (CAT. No. #K464-100, Milpitas, CA 95035, USA). The results were calculated in mg protein^−1^.

#### Indicators for brain inflammation

The brain levels of the phospho-nuclear factor kappa B p65 (p-NF-κBp65) and its downstream pro-inflammatory cytokine’s tumor necrosis factor-α (TNF-α) were measured using rat ELISA kits acquired from BioVision (CAT. No.#K4521-100, Milpitas, CA 95035, USA) and BT LAB (CAT. No. E0764Ra, 8050 Zürich, Switzerland), respectively according to the manufacturer’s guidelines.

#### Determination of brain p-AKT, p-CREB, and GSK3β levels

Following the instructions’ manufacture, the levels of phospho-protein kinase (p-AKT), phospho-cAMP-response element binding protein (p-CREB), and glycogen synthase kinase-3 beta (GSK3β) were evaluated in brain tissue homogenate using rat ELISA assay kits supplied from Sunlong Biotech Co. LTD (CAT. No. SL1158Ra, Hangzhou, China), Bioassay Techology Labolatory (CAT. No. E1411Ra, Yangpu Dist. Shanghai, China), and Fine Test^®^ (CAT. No. ER0060, Wuhan, China) respectively. The data was presented in mg protein^−1^.

### Molecular study (quantification of brain BDNF gene expression)

The RNA content of brain specimens was secluded by Direct-zol RNA Miniprep Plus (Catalogue no. R2072) purchased from ZYMO RESEARCH CORP. USA, and the amount and quality were checked by a Beckman dual spectrophotometer, USA. For the reverse transcription step, Thermo Fisher Scientific’s SuperScript IV One-Step RT-PCR kit (Catalogue no. 12594100, Waltham, MA, USA) was utilized. In a thermal profile, a 96-well plate StepOne instrument (Applied Biosystem, USA) was employed as follows: 10 minutes at 45° C for reverse transcription, 2 minutes at 98° C for RT inactivation, and initial denaturation by 40 cycles of 10 seconds at 98°C, 10 seconds at 55°C, and 30 seconds at 72°C for the amplification phase. Following the RT-PCR, the readings were computed as Cycle threshold (Ct). The fold change of the studied gene is calculated using the 2^−∆∆Ct^ method. The primer sequences were; forward 5′- CTTCCAGCATCTGTTGGGGAGACG − 3′ and reverse 5′- CACGCTCTCCAGAGTCCCATG − 3’ −3′ for BDNF (XM_032903863.1), and forward 5’- CCTCGTCTCATAGACAAGATGGT − 3’ and reverse 5’- GGGTAGAGTCATACTGGAACATG − 3’ for housekeeping gene; GAPDH (NM_001394060.2).

### Histopathological investigations

#### Hematoxylin and eosin stain

The brain tissue samples fixed in 10% neutral buffered formalin were washed, dehydrated, cleared and embedded in paraffin. Then sectioned at 5-micron thickness and stained with Hematoxylin and Eosin for histopathological examination (Bancroft and Gamble 2008). The examination of stained slides was done using a light microscope (Olympus BX50, Japan).

#### Scoring of histopathological lesion

The alterations in Hematoxylin and Eosin brain stained sections were graded as follows: no changes (0), mild (1), moderate (2), and severe (3). The scoring was evaluated as follows: <30% alterations (mild), < 30 − 50% alterations (moderate), and > 50% alterations (severe) (Baraka et al. 2023).

#### Immunohistochemistry

The immunohistochemical studies were conducted according to previous method (Saleh et al. 2022). Deparaffinized brain tissue sections were deparaffinized in xylene and rehydrated in graded alcohol. To inhibit endogenous peroxidase activity, Hydrogen Peroxide Block (Thermo Scientific, USA) was used. Antigen retrieval was accomplished by placing prepared tissue pieces in a microwave oven for 10 min with 10 mM citrate. Sections were treated for 2 h with one of the following primary antibodies: rabbit monoclonal anti-caspase-3 antibody (ab4051; Abcam, Cambridge, UK) at a dose of 1:1000 and rabbit monoclonal anti-Tau (phospho S396) antibody [E178] (ab32057; Abcam, Cambridge, UK). The sections were washed with PBS before being incubated for 10 min with Goat anti-rat IgG H & L (HRP) (ab205718; Abcam, Cambridge, UK). The sections were washed with PBS before being incubated for 10 min with Goat anti-rat IgG H & L (HRP) (ab205718; Abcam, Cambridge, UK). PBS was used to rinse the sections once more. Finally, the sections were exposed to 3, 3’-diaminobenzidine tetrahydrochloride (DAB, Sigma). The slides were mounted after being counterstained with hematoxylin. For negative controls, primary antibodies were substituted with PBS.

##### Evaluation of caspase-3 and p*-* Tau immunostaining

Five brain sections were investigated to assess the quantitative immunoreactivity of caspase-3 and p-Tau in each group (El-Maksoud et al. 2020; Shamseldean et al. 2022), Immunoreactivity was determined in 10 microscopical fields per section using a high-powered microscopic field (x 400). Color deconvolution picture J 1.52 p software (Wayne Rasband, National Institutes of Health, USA) was used to calculate the proportion of positively stained cells (%).

### Statistical analysis

The Shapiro-Wilk’s test for normality (*p* > 0.05) validated the normal distribution of data. One-way analysis of variance (ANOVA) was applied to determine the statistical disparity between means. Tukey’s test for multiple comparisons was utilized to evaluate statistical significance across distinct groups, in cases of significant F-ratio and post hoc, at *p* < 0.05 degree of significance. The graphs in this study were created with the GraphPad Prism software (version 6.0 for Windows, GraphPad Inc., San Diego, CA). Furthermore, the structures of compounds have been constructed by the aid of ChemBioDraw Ultra 14.0. The *in vivo* study’s statistical analyses were carried out using the Statistical Package for Social Science (SPSS) version 17.0 (SPSS Inc., Chicago, IL, USA).

## Results

### Phytochemical investigation

#### HPLC for natural pigment content

The ethanolic extracts of *C. aurantium* unripe fruit and leaves were subjected to HPLC analysis. The results revealed that *C. aurantium* unripe fruit implied higher contents of Chlorophyll B while the leaves have a high yield of both Chlorophyll A and total carotenoid Table 1.

**Table 1:** Total natural pigment content for *C. Aurantium* unripe fruits and leaves

Sample	Chlorophyll A	Chlorophyll B	Total Carotenoids
	(mg/100 g)		
***C.****aurantium* unripe fruit	81.37	22.97	41.33
***C.****aurantium* leaves	93.83	10.6	125.18

####  UPLC/MS/MS analysis of *C.* *aurantium* ethanol unripe fruits and leaves extracts

Fifty-seven chromatographic peaks were identified by UPLC/MS/MS (Table 2) belonging to various metabolite classes in *C. aurantium* unripe fruit and leaf extracts. By comparing different chromatographic peaks to the previous published data flavonoids represented the major metabolites identified and their fragmentation patterns could be annotated (Table 2).

**Table 2:** UPLC/MS/MS of *C. aurantium* ethanol unripe fruits and leaves

NO	Rt.	Leaves	Fruits		Molecular Formula	MS/MS Fragments	Identification	Chemical class
		Parent ion m/z (-ve)	Parent ion m/z(-ve)	Parent ion m/z (+ ve)				
1	0.99	191	191	-	C_7_H_12_O_6_	173,147,111,93	Quinic acid	Cyclitol acid
2	1.03	191	191	-	C_6_H_8_O_7_	173,129,111,87	Citric acid	Organic acid
3	1.08	-	205	-	C_11_H_10_O_4_	187,173,143,131,111,87	5,7-Dimethoxy coumarin (Citropten)	Coumarin
4	1.11	-	133	-	C_4_H_6_O_5_	115,71	Maleic acid	Organic acid
5	1.16	-	173	-	C_7_H_10_O_5_	155,111,93	Shikimic acid	Cyclohexane carboxylic acid
6	1.19	-	179	-	C_6_H_12_O_6_	161,143,125,71	Glucose	Polysaccharide
7	1.23	179	179	-	C_9_H_8_O_4_	161, 135, 89,101	Caffeic acid	Phenolic acid
8	1.29	163	163	-	C_9_H_8_O	145, 119	P-Coumaric acid	Phenolic acid
9	1.53	-	137	-	C_7_H_6_O_3_	93,65	P-Hydroxybenzoic acid	Phenolic acid
10	1.7	-	167	-	C_8_H_8_O_4_	152, 108,124	Vanillic acid	Phenolic acid
11	1.82	341	341	-	C_15_H_18_O_9_	179, 161, 135	Caffeoyl hexoside	Phenolic acid derivative
12	1.91	-	353	355	C_16_H_18_O_9_	191, 179,161, 135	Chlorogenic acid	Phenolic acid
13	2.42	341	341	-	C_12_H_22_O_11_	179,161,143	Sucrose	Polysaccharide
14	2.47	357	357	-	C_16_H_18_O_8_	195,173,136	Dihydroferuloyl-o-glucoside	Phenolic acid derivative
15	2.48	337	337	-	C_16_H_18_O_8_	191, 163	P-Coumaroylquinic acid	Phenolic acid
16	2.52	193	193	-	C_10_H_10_O_4_	178,149, 134	Ferulic a cid	Phenolic acid
17	3.21	367	367	-	C_17_H_20_O_9_	191, 173	Feruloylquinic acid	Phenolic acid derivatives
18	3.63	-	339	-	C_15_H_16_O_9_	177, 133	Aesculin	Coumarin glucoside
19	3.45	447			C_21_H_20_O_11_	357,327,297	Orientin	Flavonoid
20	4.33	447			C_21_H_20_O_11_	357,339,327,297	Isoorientin	Flavonoid
21	6.04	577			C_27_H_30_O_14_	457,413,311,293	Vitexin-2’-O-rhamnoside	Flavonoid
22	6.06	433	433	-	C_21_H_22_O_10_	387,271,151	Naringenin-O- hexoside	Flavonoid
23	6.18	609	609	-	C_27_H_30_O_16_	301,271,255,179,151	Rutin	Flavonoid
24	6.19	431	431	-	C_21_H_20_O_10_	341,311,293	Vitexin	C-Flavonoid
25	6.23	529	529	-	C_26_H_26_O_12_	367, 353,193	Dicaffeoylquinic acid methyl ester isomer	Phenolic acid derivatives
26	6.3	-	151	-	C_8_H_8_O_3_	136,92	Vanillin	Phenolic acid
27	6.49	463	463	-	C_21_H_19_O_12_	301, 271, 255	Quercetin-O- hexoside	Flavonoid
28	6.65	577			C_27_H_30_O_14_	457,413,341,323,293	Isovitexin-2’-O-rhamnoside	Flavonoid
29	6.91	-	593	-	C_27_H_30_O_15_	285, 255, 227	Kaempferol-O-rutinoside	Flavonoid
30	6.93	447	447	-	C_21_H_20_O_11_	401,285, 267, 255,	Luteolin-O- hexoside	Flavonoid
31	7.04	431			C_21_H_20_O_10_	413,341,311	Isovitexin	Flavonoid
32	7.34	431			C_21_H_20_O_10_	341,311,269	Apigenin − 7- glucoside	Flavonoid
33	7.5 (+)	609	609	611	C_28_H_34_O_15_	303,465,179,153	Hesperidin	Flavonoid
34	7.96		-	179	C_9_H_6_O_4_	161,133,119	7,8-Dihydroxycoumarin (Daphnetin)	Coumarin
35	8.72	-	355	-	C_16_H_20_O_9_	287,219,193	Ferulic acid-o- hexoside	Phenolic acid derivatives
36	8.87	327			C_18_H_32_O_5_	309,229,211,171	Trihydroxyoctadecadienoic acid	Fatty acid
37	9.94	-	-	329	C_18_H_16_O_6_	314,299,271.153	4’-Hydroxy-5,6,7-trimethoxyflavone	Poly methoxylated flavonoids
38	9.12	301	301	-	C_15_H_10_O_7_	273,255, 151,179	Quercetin	Flavonoid
39	10.2	207	207	-	C_11_H_12_O_4_	179,135,161	Ethyl caffeate	Flavonoid
40	10.23	299	299	-	C_16_H_12_O_6_	284,137	Chrysoeriol	Flavonoid
41	10.31		-	177	C_10_H_8_O_3_	162,149.133	7-Hydroxy 4-methyl coumarin (Hymecromone)	Coumarin
42	10.4	245			C_14_H_14_O_4_	227,175	Marmesin	Coumarin
43	10.56	345	345	-	C_17_H_14_O_8_	330,315,276	Limocitrin	Flavonoid
44	10.9	-	315	-	C_16_H_12_O_7_	300, 269,246	Isorhamnetin	Flavonoid
45	12.03		-	207	C_11_H_10_O_4_	192,164,149,121	6,7-Dimethylcoumarin	Coumarin
46	12.07		-	389	C_20_H_20_O_8_	374,359,341,227	6-Hydroxy 3’,4’5,7,8 Pentamethoxyflavone	Polymethoxy flavonoid
47	12.39		-	217	C_12_H_8_O_4_	202,174	5-Methoxypsoralen	Coumarin
48	12.5		-	359	C_19_H_18_O_7_	344,329,314	Demethyl Sinensetin (5-Hydroxy 3’,4’,6,7-tetramethoxyflavone)	Polymethoxy flavonoid
49	12.75		-	373	C_20_H_20_O_7_	358,357,343,329,312	Sinensetin	Polymethoxy flavonoid
50	13.48		-	313	C_18_H_16_O_5_	298,297,270,269,255	5,7,4’-Trimethoxy flavone	Polymethoxy Flavonoid
51	13.89		-	343	C_19_H_18_O_6_	328,313,299,283,282	4’,5,6,7-Tetramethoxyflavone	Polymethoxy Flavonoid
52	12.67		-	373	C_20_H_20_O_7_	343,358,328,313	(Tangeretin) 4′,5,6,7,8-Pentamethoxyflavone	Polymethoxy Flavonoid
53	14.84		-	373	C_20_H_20_O_7_	343,358,357,315	Isosinensetin	Polymethoxy flavonoid
54	12.07		-	389	C_20_H_20_O_8_	374,359,344,326	5-demethylnobiletin	Polymethoxy flavonoid
55	14.05		-	403	C_21_H_22_O_8_	388,373,211,183,163	Nobiletin	Polymethoxy Flavonoid
56	14.82		-	419	C_21_H_22_O_9_	404,389,374,371,328	Natsudaidain	Polymethoxy Flavonoid
57	15.27		-	433	C_22_H_24_O_9_	418,403,385,211	Heptamethoxyflavone	Polymethoxy Flavonoid

*Rt.; Retention time

Fifty-seven chromatographic peaks were identified by UPLC/MS (Table 2) belonging to various metabolite classes in *C. aurantium.*

Forty-nine compounds were present in fruit extract and twenty-nine peaks were identified in *C. aurantium* leaves extract by comparing different chromatographic peaks to the previous published data flavonoids represented the major metabolites identified and their fragmentation patterns could be annotated. Flavonoids, phenolic acids and their derivatives which represented the major identified compounds ionized mainly in the negative ion mode while other compounds as methoxylated flavonoids and coumarins ionized in the positive mode.

The compounds ionized mainly in the negative ion mode represented 31 compounds that gave fragments from [M-H]- ion, 16 compounds ionized in the positive ion mode gave fragments from [M + H]^+^ ion and 2 compounds were identified in the two modes.

Fruit and leaf extracts have twenty compounds common while nine compounds present in leaves extract only.

Briefly, the characteristic fragmentation ions of O-glycosides were revealed through the loss of (162 amu) represented hexose (glucose or galactose) or loss of (146 amu) for deoxysugar (rhamnose) or loss of (132 amu) for pentose (arabinose or xylose) while fragmentation ions of C-glycosides as Vitexin peak (21) whose molecular weight appear at (431)^−^ in negative mode characterized by dehydration and (0,2) or (0,3)-ring cleavages (Farag et al. 2016), peak 341 appear due to ring cleavage (M-H-90), peak 311 appear due to ring cleavage (M-H-120) (El-Sayed et al. 2017).

Briefly, the fragmentation patterns annotated for flavonoids O-glycosides were revealed through the loss of hexose as glucose or galactose (162 amu) or loss of deoxysugar as rhamnose (146 amu) or loss of pentose arabinose or xylose (132 amu). For example, quercetin and quercetin derivatives, m/z 301 represented a mass spectrum of the specific fragment ions which characterized quercetin aglycone while peak (24) has been tentatively identified as quercetin-O-hexoside m/z 463 and on the other hand peak (20) identified as rutin (quercetin -O-rutinoside) due to the loss of moieties like hexoside, pentoside or rutinoside (162, 132, 308 amu), respectively (Kumar et al. 2017).

Peak (43) nobiletin (5,6,7,8,3’,4’-Hexamethoxy flavone) molecular weight in positive mode is (403) ^+^ peak 388 appeared due to loss of methyl group (M + H-CH_3_), peak 373 due to loss of CH_2_O group (M + H-CH_2_O), peak 211(RDA, ring A), peak 183(211-CO) and peak163 (RDA, ring B) (Soares et al. 2020).

Peaks (3,18,28,33,36,38) showed six coumarins at molecular ion [M − H]^−^ at m/z identified as (Citropten), aesculin, daphnetin, hymecromone, 6,7-dimethylcoumarin and 5-Methoxypsoralen respectively, for example, 339 gave mass product ion m/z 177 characteristics to the loss of glucose (162 amu) and m/z 133 characteristic to the loss of glucose and CO_2_ (Wang et al. 2023). It was the first identification of these compounds in the two species.

####  Isolation of compounds from *C.* *aurantium* ethanol unripe fruit extract

**Fig. 1:**
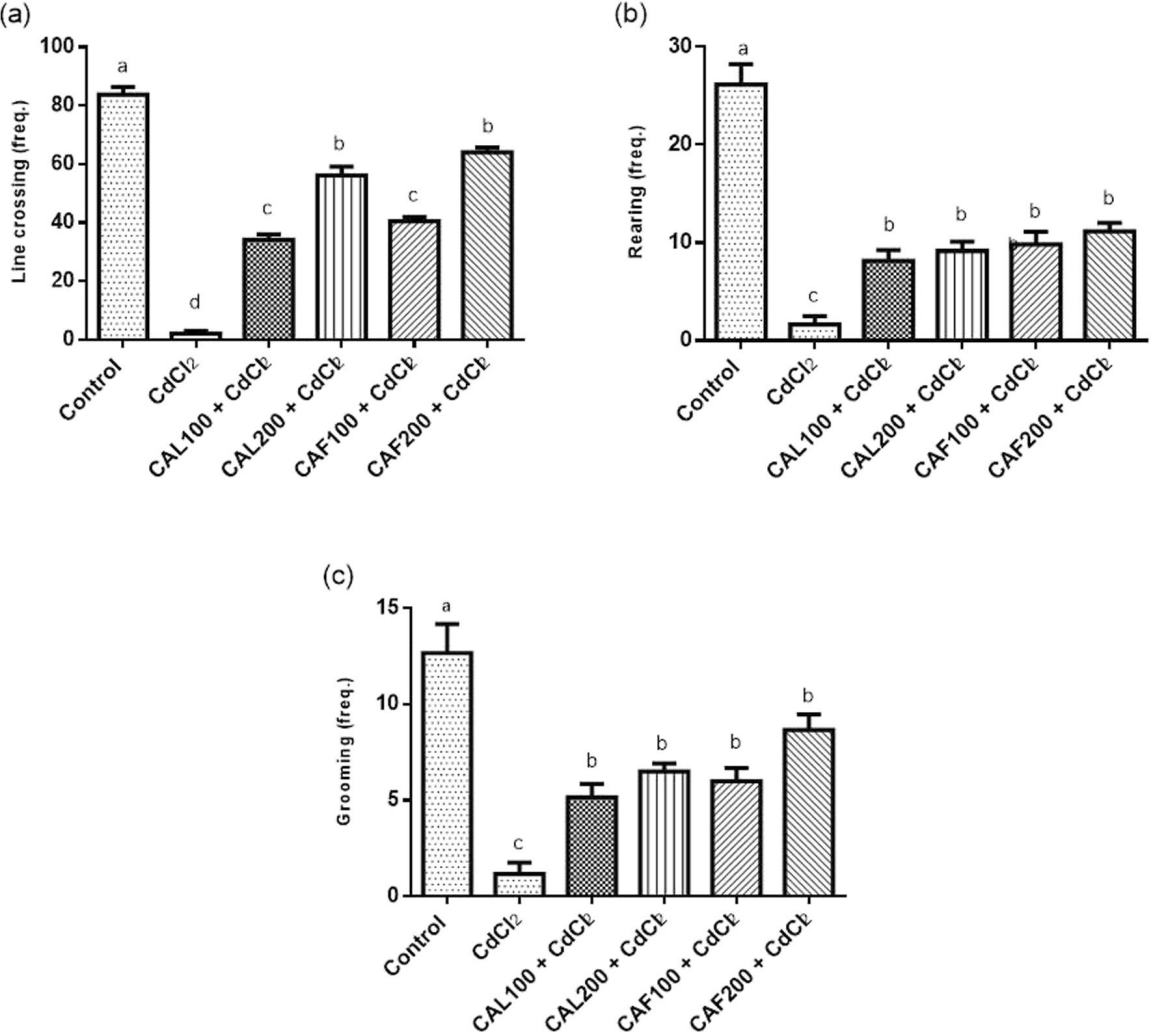
Ameliorative effects of ***C. ****aurantium *extracts on rats’ behavioral changes in the open field task. Where, (**a**) line crossing, (**b**) rearing and (**c**) grooming frequencies. Each bar represents the mean ± SE (*n* = 6) of individual group and the significance at *p* < 0.05 is illustrated by distinct letters using Tukey’s post-hoc test

Column chromatography technique on ethanol unripe fruit extract resulted in isolation of 4 compounds **(1–4)** (Fig. 1S, supplementary file). Compounds 1 and 2 were isolated from 80:20% (chloroform: methanol v/v), while compounds 3 and 4 were isolated from 50:50% (chloroform: methanol v/v). The 4 isolated compounds afforded yellow color with ammonia and different color responses with 1% AlCl_3_ solutions indicating their flavonoidal nature.

##### **Compound 1 (3’**,**4’**,**7 -Trihydroxyflavone)**

It was isolated as yellow needles (24 mg), m.p 312°C. It gave fluorescent light blue UV light 366 nm, yellow-green with 1%AlCl_3_ with R*f* 0.64. UV (λ max, nm) in MeOH: 256, 318, 340, (+ NaOMe): 267, 336, 388; +AlCl_3_: 252, 367 AlCl_3_/HCl: 256, 320; +NaOAc: 268,311, 369, NaOAc/H_3_BO_3_: 257, 313 sh 379. ^1^H-NMR (400 MHz, DMSO, δ, ppm) showed signals at 6.58(s,1H, H-3), 6.65(dd,1H, H-6), 6.68 (d,1H, H-8), 6.78(d, 1H, H-5’), 7.12 (dd,1H, H-6’), 7.36(d,1H, H-2’), 7.54(d, 1H, H-5), 12.13, 11, 01, 9.81 for the 3 OH groups. ^13^CNMR (125 MHz, DMSO δ, ppm): 147.23(C-2), 111.87(C-3), 183.10(C-4), 124.52(C-5),112.42(C-6), 168.32 (C-7), 99.14(C-8), 165.78(C-9), 114.20(C-10), 126.25(C-1’), 115.80(C-2’), 146.32(C-3’),145.86C4’), 117.93(C-5’), 122.70(C-6’). EI-MS (m/z) m/z gave M^+^ 270 for the molecular formula of C_15_H_10_O_5_ and other fragments were: 252, 243(100% rel. int.), 241,226, 124, 135,121, 108, 94.

Regarding the obtained UV data, Band I of flavones measured in methanol shows up in the range 304–350 nm. Additionally, 3’, 4’-oxygenated flavones and flavonols usually display two absorption peaks (or one maxima with a shoulder) between 250 and 275 nm, while the 4’-oxygenated equivalents have only one. The bathochromic shift noticed in Band II upon NaOMe addition indicated hydroxylation of the A-ring in flavones while the huge bathochromic shift of Band I greater than 40 nm is characteristic of the free 4’-OH group presence. Relatively, a 20 nm bathochromic shift more than 5 in bandII is remarkable for a free 7-OH group. Furthermore, another bathochromic shift on the addition of boric acid indicated the incidence of orthodihydroxyl chelation in any location of the flavonoid structure except for C-5 and 6. In this compound, a recorded acid labile complex bathochromic shift upon the addition of AlCl_3_/HCl reagent confirming the presence of ortho-dihydroxyl structure (Mabry et al. 2012). Comparing the above data with the previously reported in the literature, compound 1 was identified as 3’,4’,7 –trihydroxyflavone (Junior et al. 2008; Kim et al. 2010).

##### **Compound 2 (Isorhamnetin; 3**,**4’ **,**5**,**7-tetrahydroxy-3’-methoxyflavone)**

It was obtained as light yellow amorphous powder (21 mg), mp. 306 °C with Rf 0.68. It gave dull yellow with UV light 366 nm and dull yellow upon spraying with1%AlCl_3_ UV (λ max, nm) in MeOH: 268, 330, 368; (+ NaOMe): 279, 329 (sh), 423; + AlCl_3_: 278, 302 (sh), 426; AlCl_3_ / HCl: 272, 302 (sh), 432; +NaOAc: 269, 383; NaOAc / Boric acid: 271, 342. ^1^H-NMR (400 MHz, DMSO, δ, ppm) depicted the following peaks: 3.58 (s,1H, 3’-OCH_3_), 6.23 (d,1H, H-6), 6.51 (d,1H, H8), 6.83 (d,1H, H-5’), 7.63 (dd, 1H, H-6’), 7.71 (d, 1H, H-2’), 9.38 (s, 1H, 3-OH), 9.76 (s, 1H, 4’- OH), 10.54 (s,1H, 7-OH),12.32(s,1H, 5-OH). ^13^CNMR (125 MHz, DMSO δ, ppm): 151.12 (C-2), 136.32 (3-C), 177.16 (C-4), 164.36 (C-7), 159.24 (C-5), 98.47 (C-6), 94.10 (C-8), 158.51 (C-9), 103.32 (C-10), 122.26 (C-1’), 112.37 (C-2’), 145.93 (C-3’), 149.64 (C-4’), 115.91(C5’), 122.19 (C-6’), 56.17 (OCH_3_). EI-MS(m/z) m/z gave M^+^ 316 for the molecular formula of C_16_H_12_O_7_ and other fragments were: 301, 287, 245, 153,142, 128, 108, 69. Comparing the spectral data with previously reported in the literature, this compound can be identified as isorhamnetin (Cao et al. 2008; Manivannan and Shopna 2015).

It is to be noted that flavones and flavonols that contain hydroxyl groups at C-3 or C-5 form acid-stable complexes with boric acid while, aluminum chloride forms acid-labile complexes with flavonoids that contain orthodihydroxyl systems. No bathochromic shift occurred upon the addition of boric acid indicating the absence of orthodihydroxyl chelation and the possibility of methoxy substitution at ring B confirmed by ^1^H-NMR signal at δ 3.58 (Mabry et al. 2012).

##### **Compound 3 (Vitexin**,** Apigenin 8-C-glucoside)**

It was isolated as a light crystalline powder (23 mg), mp 249°C, Rf = 0.54. It gave purple with UV light 366 nm and light green upon spraying with1%AlCl_3_ UV (λ max, nm) in MeOH: 269, 301 sh, 339; +NaOMe: 270, 318, 388; +AlCl_3_: 268, 302, 382; +AlCl_3_/HCl: 270, 301, 386; +NaOAc 272,302,382; NaOAc/ Boric acid 269, 326sh, 348. ^1^H-NMR (400 MHz, DMSO, δ, ppm) recorded the following peaks: 3.60 (m,1H, H-6”), 3.72 (m, H, H-6”), 3.74–4.81 (m, 4H, glucose protons), 4.52(d,1H, H-1”), 6.34 (s,1H, H-6), 6.63(s, 1H, H-3), 6.74 (d, 2H, H-3`, 5`), 7.82 (d, 2H, H-2`, 6`), 10.42 (s, 1H, 4`-OH), 12.60 (s, 1H, 5-OH). ^13^CNMR (125 MHz, DMSO δ, ppm): 164.21 (C-2), 103.12 (C-3), 183.31 (C-4), 161.42 (C-5), 99.20 (C-6), 163.24 (C-7), 105.16 (C-8), 156.32(C-9), 104.41 (C-10), 122.21 (C-1`), 129.17 (C-2`, C-6`), 116.14 (C-3`, C-5`), 162.23 (C-4`), 74.51 (C-1”), 72.14 (C-2”), 79.41 (C-3”), 71.74 (C-4”), 82.21 (C-5”), 63.16 (C-6”). EI-MS (m/z) m/z gave M^+^ 432 for the molecular formula of C_21_H_20_O_10_ and other fragments were: 342, 336, 312, 284, 283, 270, 165, 152, 121, 69, and 55. Comparing the Rf with the authentic reference and the obtained analytical data with that reported in the literature, this compound can be identified as vitexin (El-Sayed et al. 2017; Eldahshan et al. 2009; Mabry et al. 2012).

##### Compound 4 (Apigenin)

It was obtained as a yellow powder, melting point: 1802°C, Rf = UV: 0.78. It gave deep purple with UV light 366 nm and light green upon spraying with 1% AlCl_3_. UV (λ max, nm) in MeOH: 266, 298 sh, 332; +NaOMe 270, 326, 390; +AlCl_3_ 269, 304, 384; AlCl_3_ /HCl 272, 300, 383; + NaOAc 269, 301, 368; NaOAc/ Boric acid: 268, 340. ^1^H-NMR (400 MHz, DMSO, δ, ppm) showed: 6.58 (s,1H, H-3) 6.62 (d,1H, H-8), 6.79 (d, 1H, H-6), 6.86(d,2H, H-3 and H-5’), 7.72 (2 H, d, H-2’ and H-6’). ^13^CNMR (125 MHz, DMSO δ, ppm): 164.18(C-2), 103.38 (C-3), 182.80 (C-4), 162.60 (C-5), 98.87 (C-6), 163.92 (C-7), 97.12 (C-8), 158.11 (C-9), 105.16 (C-10), 121.80 (C-1`), 128.86 (C-2`, C-6`), 117.32 (C-3`, C-5`), 162.16 (C-4`). EI-MS (m/z) m/z gave M^+^ 270 for the molecular formula of C_15_H_10_O_15_ while other fragments were observed at m/z 107, 117,133, 135, 151, 183, 201, and 224. This compound was identified by Rf value matching the authentic standard and the obtained spectral analyses (Fajriah et al. 2016; Riyazuddin et al. 2020).

### *In vitro* antioxidant assay

**Fig. 2:**
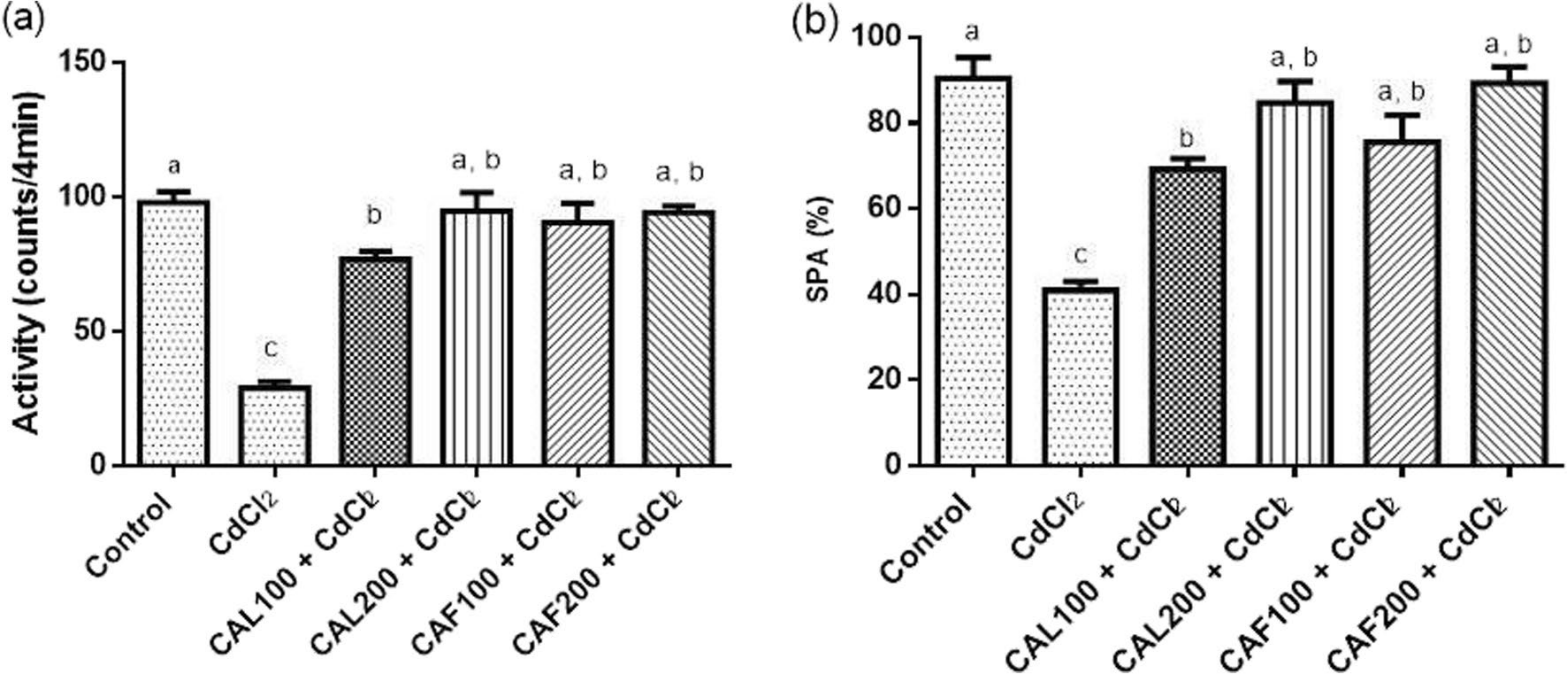
Ameliorative effects of ***C. ****aurantium *extracts on the changes of activity cage and Y maze. Where, (**a**) activity counts and (**b**) SPA% recorded in activity cage and Y maze, respectively. Each bar represents the mean ± SE (*n* = 6) of individual group and the significance at *p* < 0.05 is illustrated by distinct letters using Tukey’s post-hoc test

Figure 2Sa (supplementary file) shows the free radical scavenging activity of the two ethanolic extracts obtained from *C. aurantium* leaves and unripe fruits (CAL and CAF, respectively). The CAF demonstrated a higher antioxidant activity than CAL with an IC_50_ value of 300.57 ± 0.47, while CAL recorded IC_50_ equals 616.80 ± 0.81 (Fig. 2Sb, supplementary file). The IC_50_ of quercetin (standard) is 1.4935 ± 0.02 µg/mL.

### *In vivo* study

#### Acute toxicity study

The toxicological studies revealed that both studied extracts are non-toxic up to 2000 mg/kg bw with no mortality cases or any significant changes in rat behaviors.

#### Behavioral tasks

##### Effects of *C. aurantium* extracts on locomotion activity and exploratory behavior

Concerning open field test, intoxication with CdCl_2_ to rats exhibited a significant decline in the number of line crossing (97.41%) as well as rearing (93.63%) and grooming frequencies (90.79%) reflecting defects in locomotion ability and exploratory potentials respectively, in comparison to the untreated group. Unlike, the intra-gastric administrations of both *C. aurantium* extracts dose-dependently significantly enhanced locomotion action and exploratory changes with respect to CdCl_2_-intoxicated rats (Fig. 1).

Additionally, rats’ activity was dramatically decreased reaching 70.71% in the CdCl_2_-challenged group as reported by the data obtained from the activity cage, relative to the normal rats. By contrast, a significant improvement in rats’ locomotor activity was reported as indicated by increasing in the recorded activity counts of CAL 100 (2.63 fold), and 200 (3.24 fold) mg/kg bw, and CAF 100 (3.09 fold), and 200 (3.22 fold) mg/kg bw, which compared to the model group (Fig. 2a).

##### Effects of *C. aurantium* extracts on short-memory

As depicted in Fig. 2b, CdCl_2_-intoxicated rats demonstrated a substantial deficit in SPA% in the Y-maze experiment when compared to the control group. In contrast to the CdCl_2_ group, CAL therapy significantly improved memory disturbance at the adopted doses. It be noted that concomitant administration of CAF with CdCl_2_ to rats at both dose regimens markedly normalized SPA % compared to controls (Fig. 2b).

####  Effects of *C.* *aurantium* extracts on cadmium accumulation in brain tissue

Intoxication of rats with CdCl_2_ for 30 days resulted in a significant increment in Cd concentration in brain tissues, with respect to the untreated group as shown in Table 3. On the other hand, co-current administration of CAF or CAL significantly decreed Cd accumulation in brain tissues at the two doses level, in comparison to the CdCl_2_ group (Table 3).

**Table 3:** Ameliorative effects of *C. aurantium* extracts on cd concentration, and changes of oxidative stress indicators in rat brain tissues

Groups	Cd	8-OHdG	MDA	GSH
	(ng/g tissue)	(ng/mg protein)	(nmol/mg protein)	
Control	0.02 ± 0.01^f^	0.85 ± 0.034^e^	0.28 ± 0.018 ^e^	1.49 ± 0.03 ^a^
CdCl_2_	6.50 ± 0.21^a^	2.53 ± 0.041 ^a^	1.45 ± 0.026 ^a^	0.42 ± 0.008 ^f^
CAL100 + CdCl_2_	4.73 ± 0.04^b^	1.74 ± 0.034 ^b^	1.05 ± 0.036 ^b^	0.80 ± 0.009 ^e^
CAL200 + CdCl_2_	3.73 ± 0.04^d^	1.10 ± 0.035 ^d^	0.62 ± 0.025 ^d^	1.18 ± 0.016 ^c^
CAF100 + CdCl_2_	4.24 ± 0.02^c^	1.42 ± 0.045 ^c^	0.90 ± 0.008 ^c^	0.95 ± 0.043 ^d^
CAF200 + CdCl_2_	2.12 ± 0.01^e^	0.95 ± 0.026 ^d, e^	0.38 ± 0.025 ^e^	1.36 ± 0.017 ^b^

The results are represented as the mean ± SE (*n* = 6), and the significance at *p* < 0.05 is illustrated by distinct letters using Tukey’s post-hoc test

####  Effects of *C.* *aurantium* extracts on oxidative stress status in brain tissue

Compared to the normal rats, animals intoxicated with CdCl_2_ reported a marked elevation of brain 8-OHdg, and MDA content by 198.46% and 426.45% respectively confirming the prevalence of oxidative damage (Table 3). In contrast, oral treatment with CAL at doses 100 and 200 mg/kg succeeded in lessening the brain 8-OHdg (31.15% and 56.32%) and MDA (27.85% and 57.58%) levels, relative to the model group. Furthermore, intra-gastric administration of CAF to CdCl_2_-challenged animals significantly mitigated the increment of brain 8-OHdg and MDA by 43.93% and 37.93% respectively at the low dose, and by 62.31% and 73.96% respectively at the high dose, comparable to the CdCl_2_-group (Table 3). Similarly, a remarkable reduction in GSH content reached 71.55% in brain tissues of rats administered CdCl_2_, in comparison to the control group. Contrary to that, concurrent treatment with CAL or CAF significantly enhanced GSH levels by 1.90 fold and 2.28 fold respectively at the dose of 100 mg/kg, and by 2.80 fold and 3.21 fold respectively at the dose of 200 mg/kg, with respect to the CdCl_2_-challenged rats (Table 3).

####  Effects of *C.* *aurantium* extraxts on inflammatory mediators in brain tissue

**Fig. 3:**
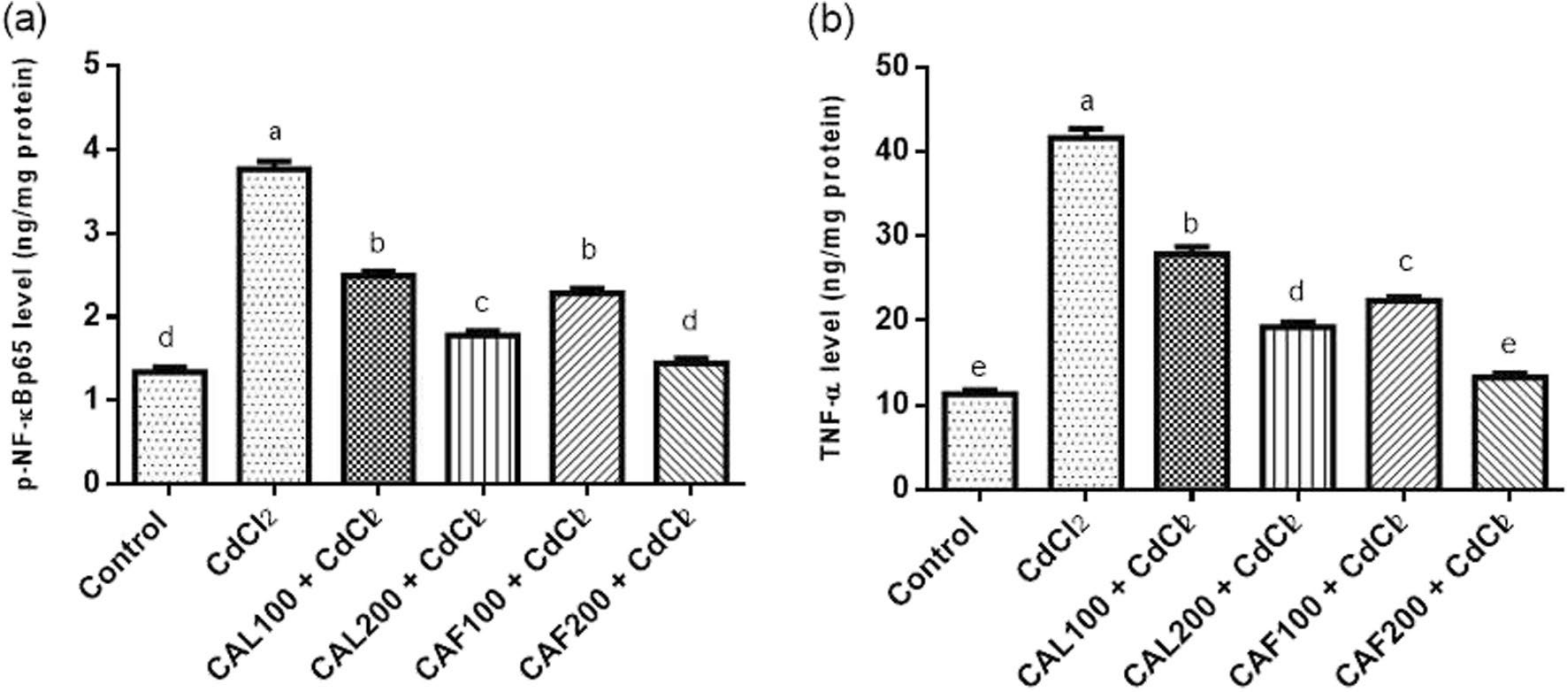
Ameliorative effects of *C. aurantium* extracts on the changes of inflammatory markers in the brain. Where, (**a**) pNF-κBp65 and (**b**) TNF-α. Each bar represents the mean ± SE (*n* = 6) of individual group and the significance at *p* < 0.05 is illustrated by distinct letters using Tukey’s post-hoc test

As depicted in Fig. 3, the rats that received a single oral dose of 5 mg/kg bw/day of CdCl_2_ for 30 consecutive days exhibited a marked activation of inflammatory reactions in the brain as demonstrated by boosting in neuronal p-NF-κBp65 (2.81 fold) and its downstream mediator; TNF-α (3.68 fold) levels, when compared to the negative control rats. Conversely, CAL treatment significantly reduced p-NF-κBp65 and TNF-α in brain tissues at the two adopted doses. Additionally, the concurrent administration of CAF at the high dose succeeded in normalizing these inflammatory markers (Fig. 3).

#### Effects of *C. aurantium* extracts on BDNF gene, p-AKT, p-CREB and GSK3β levels in brain tissue

**Fig. 4:**
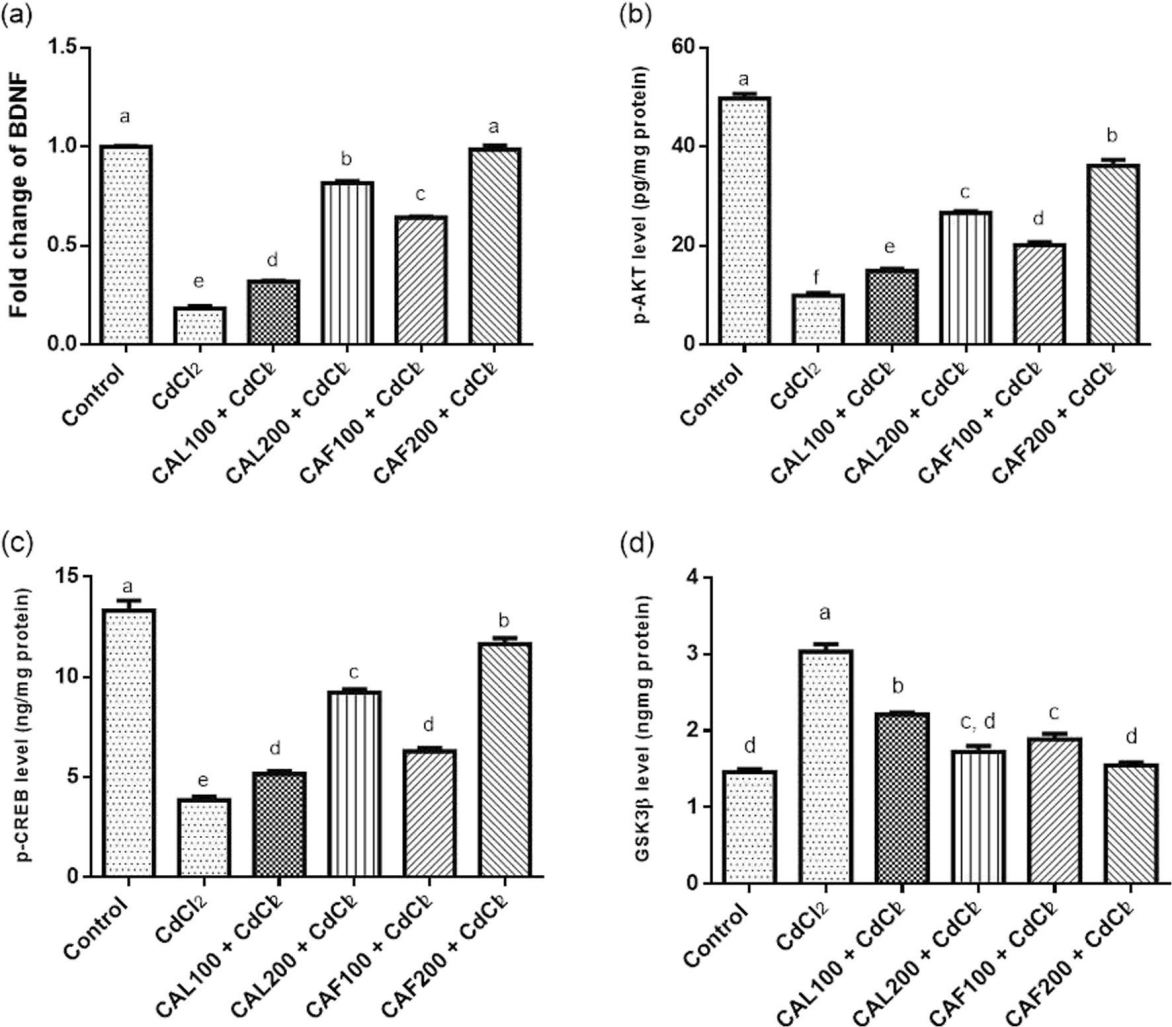
Ameliorative effects of *C. aurantium* extracts on changes of brain p-Akt/p-CREB/BDNF/GSK3β signaling pathway. Where, (**a**) BDNF gene expression, (**b**) p-AKT, (**c**) p-CREB and (**d**) GSK3β. Each bar represents the mean ± SE (*n* = 6) of individual group and the significance at *p* < 0.05 is illustrated by distinct letters using Tukey’s post-hoc test

An evident downregulation of the brain BDNF gene expression, p-AKT, and p-CREB levels, along with marked rise in brain GSK3β content were noticed in the CdCl_2_-intoxicated group, compared to the untreated control rats. Meanwhile, rats treated with CAL or CAF showed significant improvements in the aforementioned parameters at the two dose regimens. To be noted, oral administration of CAF at 200 mg/kg bw to the CdCl_2_-challenged rats significantly regulated the levels of BDNF mRNA and GSK3β in brain tissues reaching normal levels as manifested in Fig. 4.

#### Histopathological findings

##### Hematoxylin and eosin stain

**Fig. 5:**
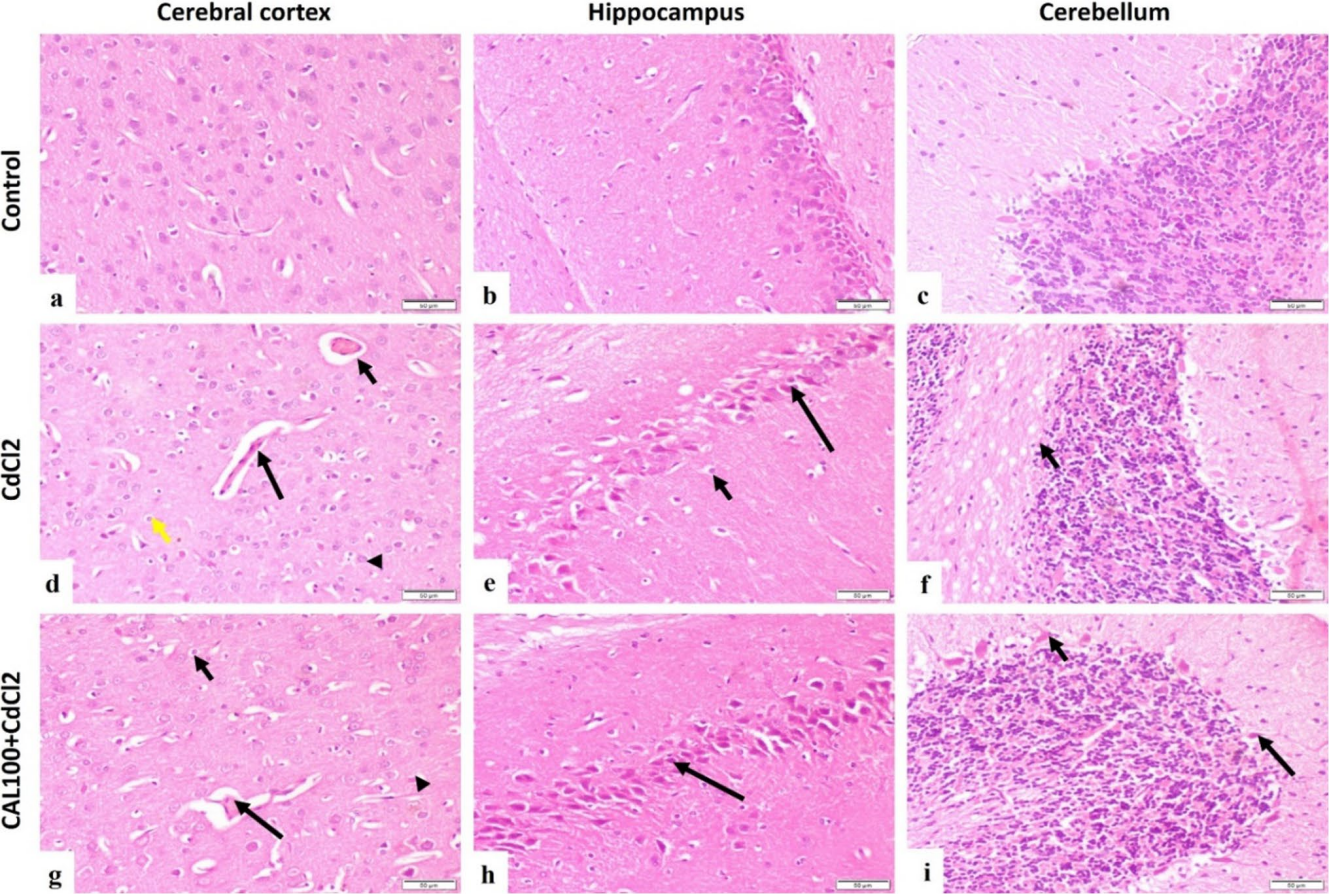
Photomicrograph, rat brain, (H&E, scale bar 50 μm).** (a-c)** cerebral cortex, hippocampus and cerebellum of control group showing normal histological structure. **(d)** CdCl_2_ group cerebral cortex showing capillary congestion (short arrow), capillary endothelial proliferation (long arrow), neuronal degeneration with neuronophagia (arrowhead) and astrocytosis (yellow arrow). **(e)** CdCl_2_ group hippocampus showing pyramidal neurons degeneration (long arrow) and astrocytosis (short arrow). **(f)** CdCl_2_ group cerebellum showing degeneration of purkinje cells (arrow). **(g)** CAL100 + CdCl_2_ group cerebral cortex showing moderately congested cerebral capillary (long arrow), astrocytosis (short arrow) and few degenerated neurons (arrowhead). **(h)** CAL100 + CdCl_2_ group hippocampus showing few degenerated pyramidal neurons (arrow). **(i)** CAL100 + CdCl_2_ group cerebellum showing degenerated purkinje cells (long arrow) and normal purkinje cells (short arrow)

**Fig. 6:**
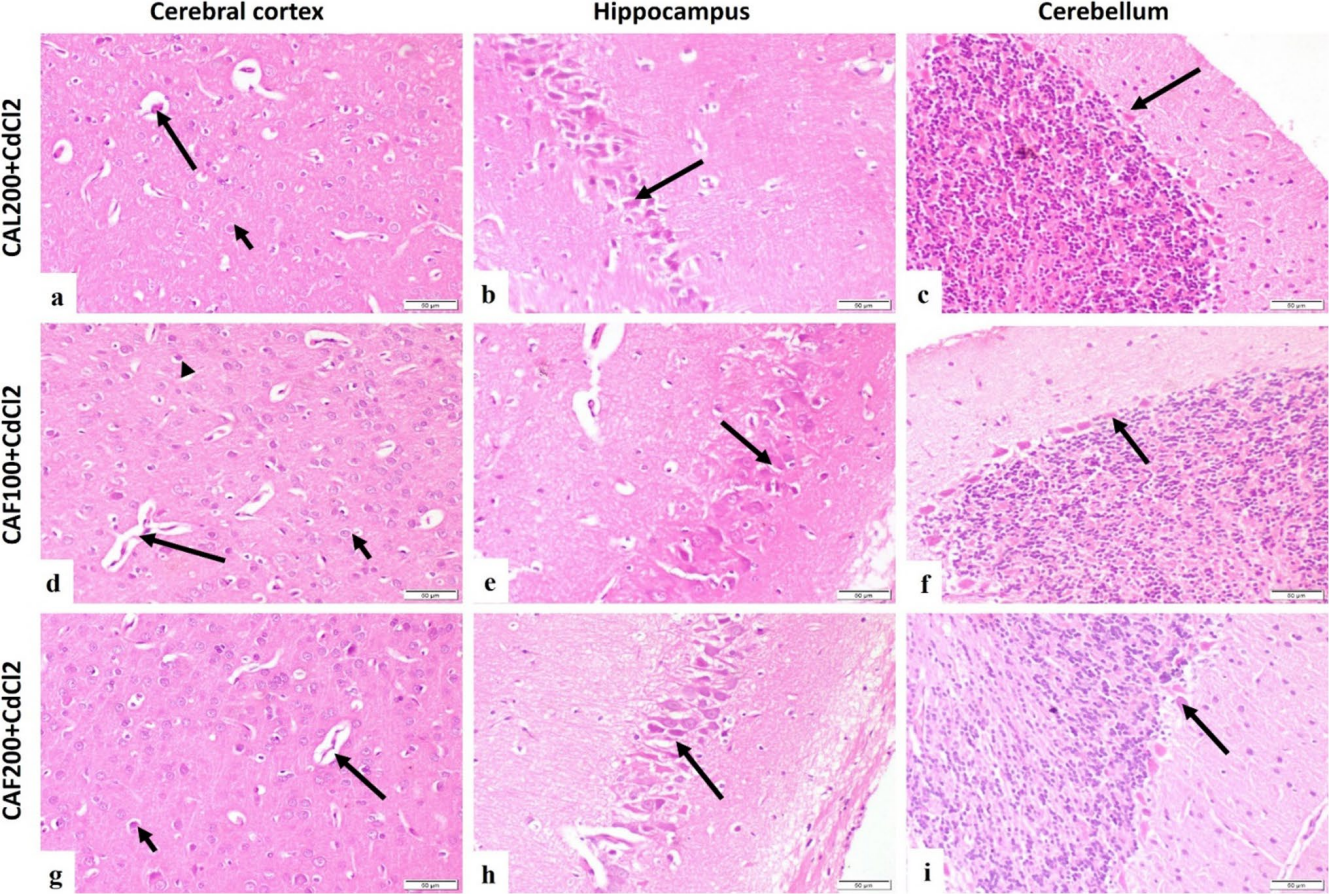
Photomicrograph, rat brain, (H&E, scale bar 50 μm).** (a)** CAL200 + CdCl_2_ group cerebral cortex showing moderately congested capillary (long arrow) and astrocytosis (short arrow). **(b)** CAL200 + CdCl_2_ group hippocampus showing nearly normal neurons (arrow). **(c)** CAL200 + CdCl_2_ group cerebellum showing normal purkinje cells (arrow). **(d)** CAF100 + CdCl_2_ group cerebral cortex showing mild capillary proliferation (long arrow), astrocytosis (short arrow) and neuronal degeneration (arrowhead). **(e)** CAF100 + CdCl_2_ group hippocampus showing few degenerated neurons (arrow). **(f)** CAF100 + CdCl_2_ group cerebellum showing nearly normal purkinje cells (arrow). **(g)** CAF200 + CdCl_2_ group cerebral cortex showing normal cerebral capillary (long arrow) and few degenerated neurons (short arrow). **(h)** CAF200 + CdCl_2_ group hippocampus showing normal neurons (arrow). **(i)** CAF200 + CdCl_2_ group cerebellum showing normal purkinje cells (arrow)

The control group demonstrated a normal histological structure of the cerebral cortex, hippocampus and cerebellum (Fig. 5a-c). Concerning the CdCl_2_ group, there were obvious changes in the cerebral cortex, hippocampus, and cerebellum. The cerebral cortex showed capillary congestion, capillary endothelial proliferation, neuronal degeneration with neuronophagia and astrocytosis (Fig. 5d). At the same time, the hippocampus of this group showed pyramidal neurons degeneration, decreased neuronal population, and astrocytosis (Fig. 5e). Moreover, the cerebellum showed degeneration of purkinje cells (Fig. 5f). The cerebral cortex of the CAL100 + CdCl_2_ group showed moderately congested cerebral capillaries, astrocytosis, and few degenerated neurons (Fig. 5g). Similarly, the hippocampus showed few degenerated pyramidal neurons (Fig. 5h), and the cerebellum showed few degenerated purkinje cells (Fig. 5i). The cerebral cortex of the CAL200 + CdCl_2_ group showed moderately congested capillaries, nearly normal neurons, and astrocytosis (Fig. 6a), and the hippocampus showed nearly normal neurons (Fig. 6b). Additionally, the cerebellum of the CAL200 + CdCl_2_ group showed normal purkinje cells (Fig. 6c). The cerebral cortex of the CAF100 + CdCl_2_ group showed mild capillary endothelial proliferation, astrocytosis, and mild neuronal degeneration (Fig. 6d). The hippocampus of the CAF100 + CdCl_2_ group showed few degenerated neurons (Fig. 6e). At the same time, the cerebellum of the CAF100 + CdCl_2_ group showed nearly normal purkinje cells (Fig. 6f). The cerebral cortex of the CAF200 + CdCl_2_ group showed normal cerebral capillaries with few degenerated neurons (Fig. 6g). Furthermore, the hippocampus of the CAF200 + CdCl_2_ group showed nearly normal neurons (Fig. 6h). Similarly, the cerebellum of the CAF200 + CdCl_2_ group showed normal purkinje cells (Fig. 6i).

##### Histopathological lesion score

All the alterations of the brain tissues were recorded, and scored according to their severity (Table 4).

**Table 4:** Histopathological alterations in brain of treated groups

Lesions	Control	CdCl_2_	CAL100 + CdCl_2_	CAL200 + CdCl_2_	CAF100 + CdCl_2_	CAF200 + CdCl_2_
- Cerebral cortex						
Capillary congestion	0	2	2	2	1	0
Capillary endothelial proliferation	0	3	2	1	1	0
Neuronal degeneration with neuronophagia	0	3	2	0	1	1
Astrocytosis	0	2	1	1	1	1
-Hippocampus						
Degenerated neurons	0	3	1	0	1	0
Decreased pyramidal neuronal cells population	0	2	2	0	1	0
Astrocytosis	0	2	1	1	1	1
- Cerebellum						
Degenerated purkinje cells	0	2	1	0	0	0

* 0 = no lesion, 1= (< 30%), 2= (< 30 – 50%), 3= (> 50%), (*n* = 5)

##### Immunohistochemical findings for caspase-3 and *p*-Tau in brain tissue

**Fig. 7:**
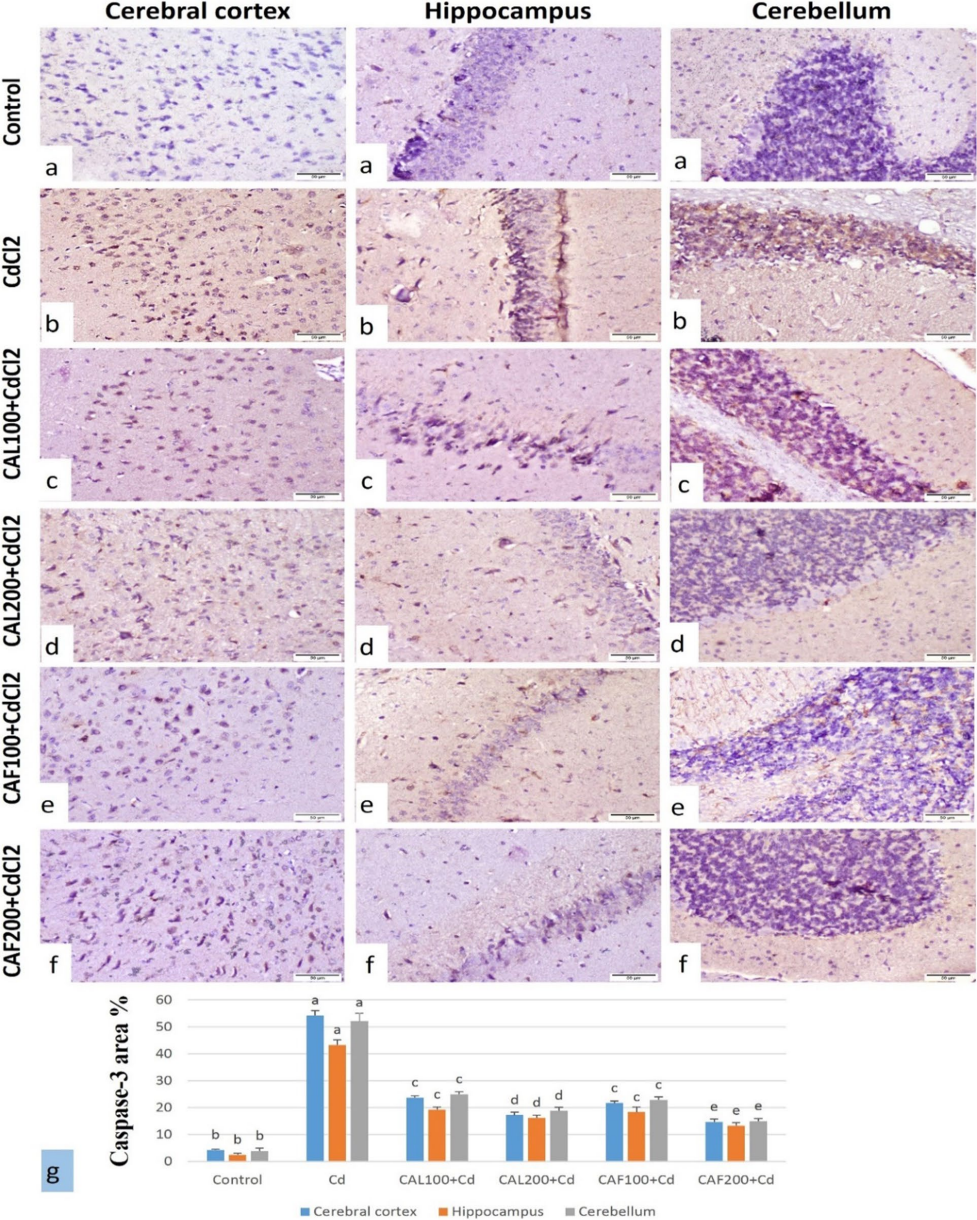
Immunostaining of caspase-3 of rat brain (Caspase-3, scale bar 50 μm).** (a)** Cerebral cortex, hippocampus and cerebellum of control group showing very weak to nil immuno-reaction. **(b)** CdCl_2_ group cerebral cortex, hippocampus and cerebellum showing strong immune-expression. **(c)** CAL100 + CdCl_2_ group showing moderate to weak positive immuno-reaction. **(d)** CAL200 + CdCl_2_ group showing weak immune-reactive cells. **(e)** CAF100 + CdCl_2_ group showing moderate to weak immune-expression. **(f)** CAF200 + CdCl_2_ group showing weak caspase-3 expression. **(g)** caspase-3 expression % area in brain tissue of different treated groups (Data was expressed as mean ± SE, dissimilar letters designating significant differences at *p* < 0.05)

**Fig. 8:**
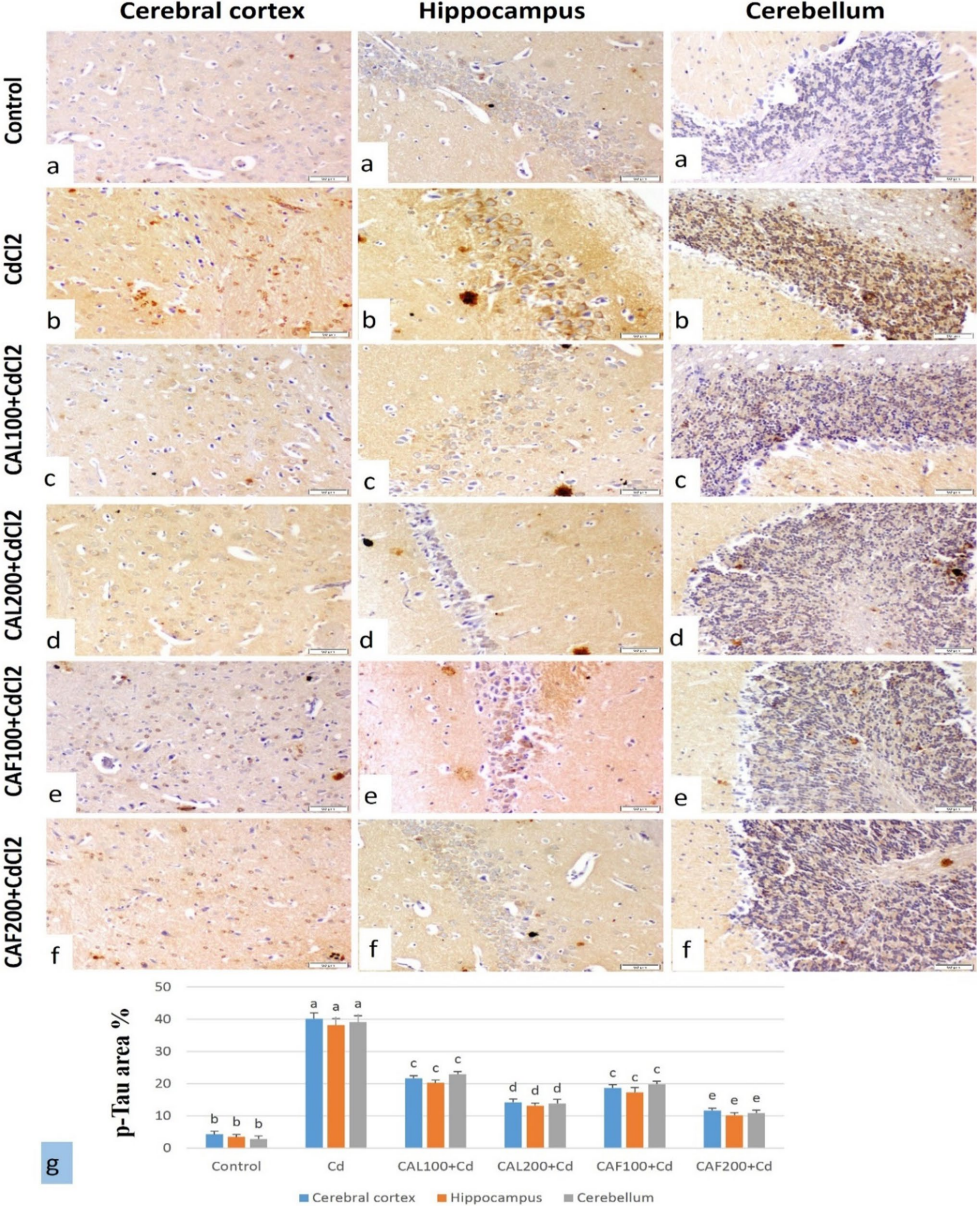
Immunostaining of p-Tau protein of rat brain (p-Tau, scale bar 50 μm).** (a)** Cerebral cortex, hippocampus and cerebellum of control group showing very weak to no immuno-reactive cells. **(b)** CdCl_2_ group showing strong immune-expression in cerebral cortex, hippocampus and cerebellum. **(c)** CAL100 + CdCl_2_ group showing moderate positive immuno-reaction. **(d)** CAL200 + CdCl_2_ group showing moderate to weak expression. **(e)** CAF100 + CdCl_2_ group showing moderate immune-expression **(f)** CAF200 + CdCl_2_. group showing weak expression. **(g)** p-Tau expression % area in brain tissue of different treated groups (Data was expressed as mean ± SE, dissimilar letters designating significant differences at *p* < 0.05)

The immune-staining of caspase-3 and p*-*Tau % area in the brain tissues of different treated groups were illustrated in Figs. 7g and 8g. the immune-staining of caspase-3 and p-Tau in the brain revealed very weak to no immune-reactive cells in the cerebral cortex, hippocampus, and cerebellum of the control group (Figs. 7a and 8a). The CdCl_2_ group showed strong expression of caspase-3 and p-Tau in the cerebral cortex, hippocampus and cerebellum (Figs. 7b and 8b). In the group treated with CAL100 + CdCl_2_, immune-staining expression was moderate to weak positive reaction (Figs. 7c and 8c). The CAL200 + CdCl_2_ group showed weak immune-reaction of the both markers (Figs. 7d and 8d). The CAF100 + CdCl_2_ group showed moderate immune-reaction (Figs. 7e and 8e). Furthermore, the group treated with CAF200 + CdCl_2_ showed weak positive expression of the both markers (Figs. 7f and 8f).

## Discussion

Since the UPLC/MS/MS spotted light on the identification of the major chemical classes abundantly found in CAF and CAL including flavonoids, phenolic acids, polymethoxyflavonoids (PMFs), coumarins, and others, the clarification of their pharmacological properties should be noted. One of the most plentiful distributed classes in plant phytochemicals is flavonoid either in aglycone or glycosidic forms. Flavonoids fundamentally act through suppression of oxidative stress as well as their interaction with multiple gene products resulting in blocking their effect thus, triggering multiple anti-inflammatory pathways (Safe et al. 2021). Moreover, coumarins perform their antioxidant activity by forming stable free radical intermediates with the oxidizing molecules (Al-Majedy et al. 2016).

It is noteworthy that PMFs abundantly present in Citrus possess a multiplicity of pharmacological properties comprising: anti-oxidant, anti-inflammatory, neuro-protection and anti-dementia (Choi et al. 2011; Mulvihill et al. 2011). In addition, several in*-vitro* and in-vivo research have elucidated the potential pertinent biological effects of Citrus PMFs, including anticancer, anti-atherosclerosis, and neuroprotective actions (Toledo et al. 2024). Recently, studies stated that PMFs crucially ameliorate or even inhibit cognitive and motor dysfunctions in animal models. These bioactivities rely on PMFs’ antioxidant and anti-inflammatory effects, beside, their role in neuronal cell death impediment, regulating the neurotrophic signals and synaptic flexibility (Matsuzaki andOhizumi 2021). For instance, the neuroprotective effects of PMFs such as 5, 7-dihydroxy-3′, 4′, 5′-trimethoxyflavone and 3-hydroxy-3′, 4′, 5′-trimethoxyflavon have been reported. This significant action was ascribed to their anti-inflammatory, antioxidant, and monoaminergic properties (Singh et al. 2024a, b). On the other hand, phenolic acids and their derivatives performed their anti-inflammatory potential by quashing pro-inflammatory mediators through NF-κB inhibition (Rahman et al. 2021). In the present study, several flavonoid compounds viz. rutin, quercetin, and chrysoeriol have been identified in both extracts which possess significant neuroprotective actions (Çelik et al. 2020; Kale et al. 2018; Shao et al. 2021). As for the isolated compounds, compound 1 (3’,4’,7 –trihydroxyflavone) exerts the anti-inflammatory effect through the prohibition of TNF pathways (Wang et al. 2023). Isorhamnetin (compound 2) is originally one of the most widely known monomethoxyflavonol. Recent research reported its anti-inflammatory effect through attenuating NF-κB signaling pathway, and antioxidant properties via lowering plasma MDA levels with increasing GSH content, in addition to its anti-depression action (Alqudah et al. 2023; Gammoh et al. 2023). Furthermore, the beneficial effects of vitexin (compound 3), a C-glycosyl trihydroxyflavone, against oxidative stress-related neurodegenerative diseases were documented via exerting anti-oxidant and anti-inflammatory actions and consequently improve neurobehavioral disorders (Babaei et al. 2020). Moreover, apigenin (compound 4) was stated to diminish the neuro-inflammatory cascade process by down-regulating NF-κB phosphorylation suppressing the level of pro-inflammatory markers including TNF-α (Ginwala et al. 2021; Mulherkar et al. 2021). In addition, a recent study deduced the neuroprotective influence of apigenin via alleviating the oxidative stress occurrence in the brain tissues in a rat model of neuro-degeneration (Baraka et al. 2024).

On top of that, considerable amounts of chlorophyll A and B have been found in the examined extracts. Existing studies revealed the bio-activities of chlorophyll and its derivatives including antioxidant, anti-cancer, anti-mutagenic, and anti-genotoxic. Moreover, the neuroprotective efficacy of chlorophyll has been well documented by former investigations on some neurodegenerative disorders (Choi andLee 2018; Hannan et al. 2020; Martins et al. 2023). For instance, in a model of mice undergoing brain ischemia and reperfusion, the pretreatment with chlorophyll salt showed neuroprotective potentials by improving motor coordination and memory function. These effects were ascribed to chlorophylls’ antioxidant capacity (Rehni et al. 2007).

Environmental heavy metal toxicity has played a main role in the onset and development of CNS-related illnesses and neurodegenerative disorders around the world. The bioaccumulation of Cd, which is abundant in nature, frequently goes unnoticed until it develops into a chronic clinical condition. All age groups and organs are susceptible to Cd-induced toxicity, though the effect on the CNS is more severe and pervasive in both the developing and adult human brain. There are no known clinically effective treatments for those who have Cd poisoning (de Carvalho Machado and Dinis-Oliveira 2023). Therefore, there is an urgent need for a global response to this health issue. In this concern, a rat model of Cd-triggered neurotoxicity was established to verify the potential neuroprotective impacts of the alcoholic extracts of *C. aurantium* unripe fruit and leaves. Moreover, the DPPH assay was conducted *in vitro* to validate and compare the antioxidant capacity of the studied extract. In order to investigate the dose-response relationship, the two examined extracts were given at two doses; low dose (100 mg/kg bw), and high dose (200 mg/kg bw). To be noted, these doses were chosen based on the results obtained by the acute toxicity studies of the extracts which documented the high safety profile of these materials at a dose up to 2000 mg/kg rat bw.

Allied to several scholars, our study’s findings showed that Cd can diffuse through BBB and accumulate in the brain tissue with a significant amount leading to severe neurological consequences (Lech and Sadlik 2017; Wang and Du 2013). According to a prior study, prolonged exposure to Cd can destruct the BBB-forming cells, compromising the barrier’s integrity and build-up in brain tissue, resulting in serious neurological changes (Jaafarzadeh et al. 2022). Due to the nerve tissue’s restricted/limited capacity for antioxidants, high oxygen consumption, and enriched lipid level, Cd deposition augmented the progression of the oxidative challenge/insult in brain tissue (Treviño et al. 2022). Free Cd ions disrupt energy metabolism, membrane integrity, and mitochondrial function by attacking thiol group-containing proteins such as GSH. This leads to an increase in ROS generation and the onset of oxidative damage. ROS are produced as a result of Cd up-regulated NADPH oxidase, which upsurges cell membrane lipid peroxidation and subsequently, diminution of GSH content, stimulation the Caspases cascade, and finally DNA disintegration (Branca et al. 2020). Furthermore, Cd was reported to aggravate the level of 8-OHdG which is a key index of endogenous oxidative DNA damage (Omari Shekaftik and Nasirzadeh 2021). In agreement with these, Cd intoxication significantly increased the content of MDA, and 8-HdG which up-regulating the pro-apoptotic protein caspase-3 coupled with a marked decrease in the GSH pool in the neuronal tissue, compared to the control brain tissue.

Here, we found that co-administration of both extracts of *C. aurantium* with Cd could considerably reduce the accumulated amount of Cd, MDA and 8-HdG contents in the brain tissues, along with a rise in the brain GSH level. These results supported the antioxidant properties of both extracts with better action for unripe fruit one at the dose of 200 mg/kg rat bw as validated by the *in vitro* DPPH study. The observed ability of the studied extracts of *C. aurantium* in eliminating Cd deposition could be explained in two ways: exerting a direct action in chelating Cd ions which might be attributed to their phytochemical active constituents and/or indirectly through preserving GSH content that can chelate Cd.

The Akt/GSK-3β signal pathway is one of the main pathways involved in neurodegenerative ailments (Lei et al. 2008). In the brain, GSK-3β acts as a key switch governing numerous signaling pathways that participate in the pathophysiology of neurodegenerative disorders (Giese 2009). AKT was inferred to have an inhibitory effect on GSK-3β activity by phosphorylation. Accumulated evidence deduced that downregulation of AKT results in stimulation of GSK-3β activity which is involved in the manifestation of negative impacts, including mitochondrial dysfunction and consequently neural death due to deposition within neural cells (Freyberg et al. 2010; Zhu et al. 2010). Additionally, GSK-3β has been stated to act as a molecular regulator of tau protein phosphorylation, where the active form of GSK-3β is involved in the phosphorylation of tau protein (Takashima 2006). In diverse *in vivo* models of neurodegenerative disorders, inhibition of GSK3 through phosphorylation which in turn relieved tau hyperphosphorylation was a molecular target to improve behavioral deficits, including cognitive and memory functions in experimental animals (Ly et al. 2013; Serenó et al. 2009). Despite the essential role of tau protein in stabilizing the cytoskeleton and regulating the axonal transmit by networking with microtubules (MTs) via its C-terminal binding domain, the hyperphosphorylation of tau protein is a fundamental motive for the subsiding of its competence to cooperate with MTs. This dysfunction has been found to cause destabilization of MTs network, and ultimately neuronal death exerting a neurotoxic influence such as chromatin intensification, DNA shatter, and caspase-3 instigation, and lastly incidence of neuro-degeneration. Therefore, hyper phosphorylation of tau protein is suggested to be an initial episode for the emergence of tau pathology (Ma et al. 2021).

An additional signaling pathway that is crucial for proper brain cell function is the Akt/CREB/BDNF axis (Srivastava et al. 2018) which makes it an important target for many experimental neurological therapies. Throughout brain growth and neurogenesis, CREB acts as a significant transcription element. CREB becomes activated when it is phosphorylated, and numerous protein kinases, particularly Akt, phosphorylate this transcription factor and convert it to its active form (Meffre et al. 2015). CREB functions on DNA to increase the creation of the BDNF protein, which is necessary for neurogenesis and neuronal proliferation (Esvald et al. 2020). Furthermore, CREB signaling has lately been implicated in a number of brain pathological diseases, including cognitive and neurodegenerative illnesses, implying a key role for CREB signaling in synapse function (Yan et al. 2022). BDNF, a CREB activation indicator gene, is a critical moderator in synaptic transmission and a significant contributor in the growth, survival, and preservation of neuron activities in the CNS as it is captured by activated synapses to modify synapse structure and strength, which stabilizes memory. Therefore, reduction of the level of BDNF has been related to the progress of neuronal dysfunction and neurodegenerative disorders A substantial opposite relationship between p-CREB and GSK3β (active form) and inflammation has been demonstrated (Green andNolan 2012). In addition, prior studies have deliberated that inflammation plays a major part in the pathogenic processes that underpin neurodegenerative illnesses and diseases (Baraka et al. 2020; Glass et al. 2010). A growing amount of research suggests that GSK3β (un-phosphorylated form) triggered inflammatory response across up-regulation of NF-κB signaling pathway (Hoeflich et al. 2000; Jorge-Torres et al. 2018).

Given the importance of the discussed signaling pathways on brain functions and its vital role in the pathogenesis of neuronal ailments, we evaluated the potential impacts of Cd on parameters-related AKT/GSK3β/CREB/BDNF pathways. Our data emphasized that a significant dis-regulation in these pathways in the Cd-challenged group was noticed as demonstrated by a down-regulation in neural p-AKT and p-CREB proteins, and BDNF gene expression, coupled with a marked increase in brain GSK3β, p-NF-κBp65, and TNF-α levels, in relative to the results of normal rats. The outcomes obtained from immunohistochemical studies deduced that brain tissues of CdCl_2_-intoxicated rats showed activation of caspase-3 and hyperphosphorylation of tau protein in different brain regions, supporting the incidence of apoptosis and memory disturbance. These findings are in line with previous research investigating mechanistic aspects by which Cd could induce neurotoxicity (Namgyal et al. 2020; Srivastava et al. 2023).

In various experimental animals, the spatial working memory and cognitive ability of rats were assessed using the Y-maze test, an indicator of the systematic working of the cortex and hippocampus (Baraka et al. 2023; Oboh et al. 2020). Moreover, locomotion and exploratory behavior could be evaluated by activity cage and open field test (Baraka et al. 2020, 2023). In our experiment, marked deficits in locomotion, exploratory, cognitive, and memory functions were recorded following Cd exposure as demonstrated by the outcomes of activity cage, open field, and Y-maze tests. These results are in line with earlier research (Lamtai et al. 2018; Oboh et al. 2020) which informed that the depressogenic impact of Cd could lead to memory and cognitive dysfunction. These impairments could be highly linked to the deleterious effects of Cd on the BDNF signaling pathway along with increment of GSK3β and tau hyper phosphorylation (Shati andAlfaifi 2019). As previously mentioned in the literature, Cd toxicity exhibited physiological disorders such as Alzheimer’s like-symptoms, and anxiety and locomotion defects (Arab et al. 2023).

By contrast, findings from this study revealed that treatment with CAL or CAF dose-dependently enhanced Cd-induced neurobehavioral impairments in rats by improving rats’ activity, exploratory and memory functions. These data imply that the beneficial effects of the given extracts may be attributable to lessened Cd bioavailability in the brain, resulting in preserving brain functions. In addition, a significant up-regulation in p-AKT, p-CREB, and BDNF mRNA levels, as well as reduction in GSK3β and tau hyperphosphorylation have been noticed in rats treated with CAL or CAF in a dose-dependent manner, when compared to the CdCl_2_ group. In addition to that, CAL or CAF exhibited anti-inflammatory effects as evidenced by marked repression in p-NF-κBp65 and its downstream cytokine; TNF-α levels in brain tissue as compared to the CdCl_2_-challenged group. These neuroprotective impacts of both extracts could be referred to their diverse phytochemical constituents that possess antioxidant and anti-inflammatory activities, so they might act in a synergistic way in combating Cd toxicity. It worth mentioning that flavones are generally possess the ability to reduce highly oxidizing radicals, hence, producing a stable quinone structure (Pietta 2000).

Allied to former research, serious degenerative changes in brain regions have been observed in CdCl_2_-intoxicated rats, as demonstrated by the histopathological examinations (Afifi and Embaby 2016; Gök and Deveci 2022). For example, the cerebral cortex of this group revealed capillary congestion, capillary endothelial proliferation, neuronal degeneration with neuronophagia and astrocytosis, while the hippocampus manifested pyramidal neuron degeneration, decreased neuronal population, and astrocytosis. Meanwhile, concurrent administration of CAL or CAF significantly attenuated all these toxic impacts of Cd on different brain regions as inferred by the histopathological outcomes.

As deduced by the statistical analyses of our research’s outcomes, the best effective actions of the two examined extracts have been raised in a dose-dependent manner. Additionally, among the examined extracts, CAF showed better records with significant improvement in all evaluated parameters of the rats treated with CAF at the dose of 200 mg/kg bw.

## Conclusion

In the light of available evidence, the neuroprotective effects of *C. aurantium* extracts with a superior action of unripe fruit extract at the dose of 200 mg/kg rat bw. being rich in different phytochemical classes against Cd-induced brain injury in rats could be based on modulating multiple homeostatic mechanisms involved in neurotrophic response, oxidative stress, inflammation, apoptosis, cognition, and mood-related disorders, in particular through Akt/GSK3β or Akt/CREB/BDNF signaling pathways. These outcomes may pay to a better thoughtful of the neuroprotective role of the examined extracts especially CAF at the high dose level, underscoring the influence of this extract in the diet for human health, possibly averting brain damage linked with Cd exposure. Taken together, our results give a strong rationale for exploring the ethanolic extract of *C. aurantium* unripe fruit as a potential treatment for neurodegenerative disorders in future human studies phase trials.

## Supplementary Information

Below is the link to the electronic supplementary material.

Supplementary Material 1 (DOCX 37 KB)

Supplementary Material 2 (JPG 49 KB)

## Data Availability

Data will be made available on request.

## Abbreviations

8-OHdG: 8-hydroxydeoxyguanosine
BBB: blood-brain barrier
BDNF: brain-derived neurotropic factor
CAF: ethanolic extract of Citrus aurantium unripe fruits
CAL: ethanolic extract of Citrus aurantium unripe leaves
Cd: Cadmium
CdCl_2_: Cadmium Chloride
DPPH: 2,2-diphenyl-1-picrylhydrazyl
GSH: glutathione
GSK3β: glycogen synthase kinase 3 beta
HNO_3_: Nitric acid
MDA: Malondialdehyde
NF-κB: nuclear factor kappa B
p-Akt: phospho-protein kinase B
p-CREB: phospho- cAMP-response element binding protein
p-NF-κBp65: phospho-nuclear factor kappa B p65
ROS/RNS: reactive oxygen/nitrogen species
SPA: Spontaneous alteration
SPSS: Statistical Package for Social Science
TNF-α: tumor necrosis factor-α
