# Supplementary material for: Metabolomics analyses and comparative insight to neuroprotective potential of unripe fruits and leaves of Citrus aurantium ethanolic extracts against cadmium-induced rat brain dysfunction: involvement of oxidative stress and akt-mediated CREB/BDNF and GSK3β/NF-κB signaling pathways
Source: Metab Brain Dis. 2025 Jan 6;40(1):89. doi: 10.1007/s11011-024-01513-6 (PMC11703990; doi:10.1007/s11011-024-01513-6)
Supplement: Supplementary file 2 [file 11011_2024_1513_MOESM1_ESM.docx]

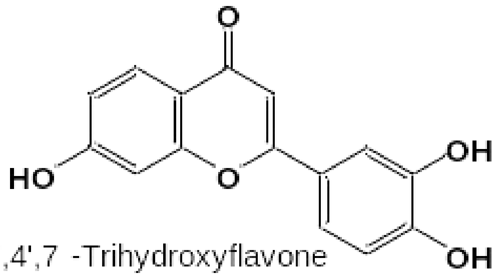

3',4',7 -Trihydroxyflavone

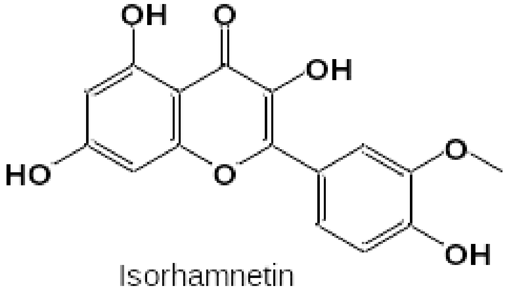

Isorhamnetin

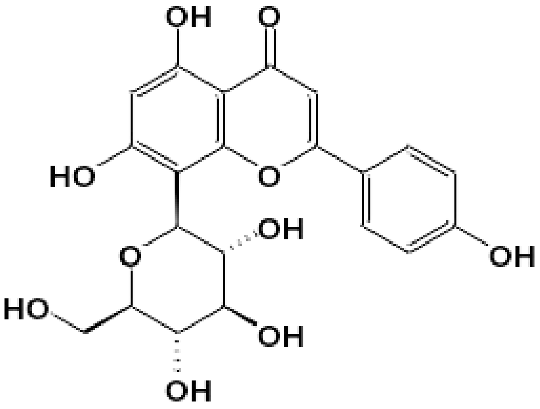

Vitexin

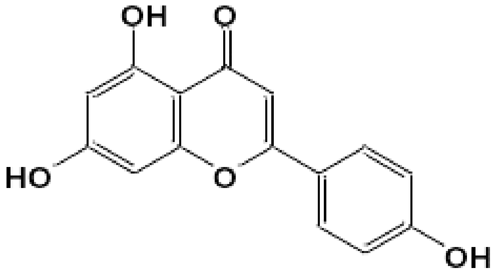

Apigenin
